# Efficacy of serum miRNA test as a non-invasive method to diagnose nonalcoholic steatohepatitis: a systematic review and meta-analysis

**DOI:** 10.1186/s12876-020-01334-8

**Published:** 2020-06-12

**Authors:** Shengliang Xin, Qiao Zhan, Xiaofan Chen, Jinghang Xu, Yanyan Yu

**Affiliations:** Department of Infectious Diseases, Peking University First Hospital, Beijing, China

**Keywords:** MicroRNA, Nonalcoholic steatohepatitis, Obesity, Body mass index

## Abstract

**Background:**

Nonalcoholic steatohepatitis (NASH) is a key turning point during the progression of nonalcoholic fatty liver disease (NAFLD). Recent studies have shown that serum miRNA tests may be effective in the diagnosis of NAFLD. We conducted a meta-analysis to assess the evidence for the diagnostic efficacy of serum miRNAs in patients with NAFLD and its subtype, NASH, in particular.

**Methods:**

After a systematic review, sensitivity, specificity, and area under the receiver operating characteristics curve (AUROC) were pooled to determine the efficacy of serum miRNA test for the diagnosis of NAFLD and NASH. Clinical utility was evaluated by Fagan’s nomogram and likelihood ratio scattergram. Heterogeneity was evaluated by subgroup analysis and meta-regression. Publication bias was detected by Deeks’ funnel plot.

**Results:**

We included 27 trials containing 1775 NAFLD patients (including simple steatosis and NASH) and 586 NASH patients. For NAFLD vs NASH, the pooled sensitivity, specificity, and AUROC were (0.71 vs. 0.74), (0.76 vs. 0.85) and (0.80 vs. 0.86), respectively. Serum miRNA had high accuracy for distinguishing NASH from simple steatosis, with an AUROC of 0.91. Among the most commonly studied serum miRNAs, miRNA-34a showed moderate diagnostic accuracy for NAFLD and the lowest heterogeneity (sensitivity *I*^*2*^ = 5.73%, specificity *I*^*2*^ = 33.16%, AUROC = 0.85). According to subgroup analysis and meta-regression, a lower BMI (< 30 kg/m^2^) might be a crucial source of heterogeneity.

**Conclusions:**

As a novel non-invasive method, serum miRNA test exhibited robust diagnostic efficacy for NASH. Among these well-studied miRNAs, miRNA-34a was more available for diagnosis. Diagnosis of NAFLD by serum miRNA is more likely to be accurate in patients with BMI ≥ 30 kg/m^2^.

## Background

Non-alcoholic fatty liver disease (NAFLD) has become a major burden among chronic liver diseases. According to the latest epidemiological studies, the prevalence of NAFLD is approximately 25% worldwide [1]. In developed countries such as the United States, the prevalence of NAFLD is 30% [1]. In developing countries such as China, the prevalence has reached up to 32.9% [2]. NAFLD comprises a spectrum of pathological conditions, including simple steatosis (NAFL), nonalcoholic steatohepatitis (NASH), fibrosis, cirrhosis and hepatocellular carcinoma (HCC). It should be noted that NASH is a crucial stage during NAFLD progression. Studies have shown that approximately one-sixth of NAFL patients progress to NASH [1], and 20% of NASH patients can develop cirrhosis [3]. Furthermore, some studies have indicated that NASH patients have a 60% greater chance of progression to HCC than that of NAFL patients [4]. The traditional view suggests that HCC formation is a multi-stage process, involving inflammation, fibrosis and cirrhosis. However, recent research found that NASH can progress to HCC without fibrosis and cirrhosis [5]. Therefore, early diagnosis of NAFLD, in particular NASH, is important.

Liver biopsy is the gold standard for diagnosis and staging of NASH; however, its clinical application is restricted by patients’ reluctance. Moreover, liver biopsy has potential complications such as bleeding, especially when patients are in decompensated conditions. Therefore, there is an urgent need to develop a non-invasive approach for diagnosis of NASH. To date, the well-studied serological biomarker is cytokeratin (CK)-18. CK-18 is an indicator of hepatocyte apoptosis. Increased CK-18 suggests severe damage in the liver parenchyma [6]. In terms of diagnostic efficacy for NASH, CK-18 shows moderate accuracy with specificity of 80% and the area under the receiver operating characteristics curve (AUROC) of 0.83, however, its sensitivity is low at 60% [7]. Other indexes such as alanine aminotransferase (ALT) and aspartate aminotransferase (AST) can give false-negatives [8]. Thus, a better non-invasive biomarker for diagnosing NASH is still needed.

MicroRNAs (miRNAs) are short, non-coding single-stranded RNAs strand of 20–25 nucleotides. miRNAs play complicated and important roles in regulating the expression of downstream genes [9]. Many experiments and clinical trials have confirmed that miRNAs are closely related to NAFLD [10–13]. miRNAs target a variety of genes related to lipid metabolism and pro-inflammatory factors, that are involved in pathogenesis of NAFLD [14]. A recent meta-analysis reported that circulating miRNA has moderate diagnostic accuracy in NAFLD [15]. However, NAFLD is a general term that consists of several pathological subtypes. Simple steatosis, fibrosis and cirrhosis can all be identified by alternative methods such as B-scan ultrasonography, computed tomography (CT) and Fibroscan [16–18]. NASH is the most hazardous stage, but cannot be diagnosed by radiological methods. Hence, there is a need to evaluate the diagnostic efficacy of serum miRNA for NASH. In this meta-analysis, we analyzed the value of serum miRNAs for diagnosis of NAFLD, in particular the subtype, NASH.

## Methods

### Literature retrieval

We have registered a review protocol on the website https://www.crd.york.ac.uk/prospero (CRD42020172385), and performed this study based on the preferred reporting items for systematic reviews and meta-analysis (PRISMA) guidelines (Additional file 1: PRISMA 2009 Checklist). All relevant articles on the application of miRNA in the diagnosis of NAFLD available on multiple electronic databases including PubMed, Science Direct, and Cochrane Library up to February 1, 2020 were searched. The retrieval strategy was (“NAFLD” OR “Non-alcoholic Fatty Liver Disease” OR “NASH” OR “Non-alcoholic Steatohepatitis”) AND (“microRNAs” OR “miRNA” OR “microRNA” OR “miR” OR “hsa-miR”). Language was not limited. Additional literature was not included in the study. We collected 3956 records according to retrieval strategy. The preliminary screening process (title and abstract screening) was performed by two authors (SLX and QZ) independently and blindly. The second screening process (full-text review) was performed by all authors. The literature was managed by Endnote X9.

### Eligibility criteria

We included articles that met the following conditions: (1) diagnostic trials; (2) trials with control and case cohorts (NAFLD or NASH); and (3) all NAFLD cases (including NAFLD and NASH) were confirmed by liver biopsy. We excluded articles with the following conditions: (1) other irrelevant liver diseases (e.g., alcoholic liver disease, viral hepatitis and drug-induced liver injury); (2) miRNAs were extracted from other tissues than serum; (3) necessary statistical data were not available for calculating the test performance parameters including true-positive (TP), false-positive (FP), true-negative (TN) and false-negative (FN) rates; and (4) duplicated records.

### Common characteristics of included trials

(1) NAFLD was confirmed when>5% of hepatocytes developed steatosis; (2) NASH was confirmed by NAFLD activity score (NAS) ≥ 5; (3) miRNA level was quantified by reverse transcription-polymerase chain reaction (RT-PCR); (4) in NAFLD trials, the control cohort comprised healthy individuals, and the case cohort comprised NAFLD patients (including NAFL and NASH); and (5) in NASH trials, the control cohort comprised individuals with NAS < 5, and the case cohort comprised NASH patients with NAS ≥ 5.

### Data extraction and literature quality assessment

We constructed a 2 × 2 contingency table and calculated the numbers of TP, FP, FN and TN results in each trial. Quality assessment of the included articles was assessed by two authors independently using the Quality Assessment of Diagnostic Accuracy Studies (QUADAS-2), as previously described [18].

### Statistical analysis

*Stata* SE version 15 was used to perform the meta-analysis. The first step was to calculate pooled statistical values, including sensitivity, specificity, positive likelihood ratio (PLR), negative likelihood ratio (NLR) and diagnostic odds ratio (DOR). *I*^*2*^ statistic was used to evaluate the heterogeneity among trials. *I*^*2*^ > 50% indicated a considerable heterogeneity, thus, random-effects model was used. The second step was to conduct summary receiver operating characteristics (SROC) curve analysis. If SROC curve had a “shoulder-arm shape”, the threshold effect was considered. AUROC values of 0.5–0.7, 0.7–0.9 and 0.9–1.0 suggest low, moderate and high diagnostic accuracy, respectively. The third step was to detect the source of heterogeneity by conducting subgroup analysis and meta-regression. For meta-regression, a covariate with *P* < 0.05 was considered the significant factor to induce heterogeneity. The fourth step was to assess publication bias. Deeks’ funnel plot was applied to examine the potential publication bias caused by any asymmetry of the trials. *P* < 0.05 for the slope coefficient indicated test asymmetry and suggested significant publication bias [19]. Finally, we used Fagan’s nomogram and a likelihood ratio scattergram to evaluate the clinical utility of this method.

## Results

### Literature retrieval, characteristics and quality assessment

**Fig. 1:**
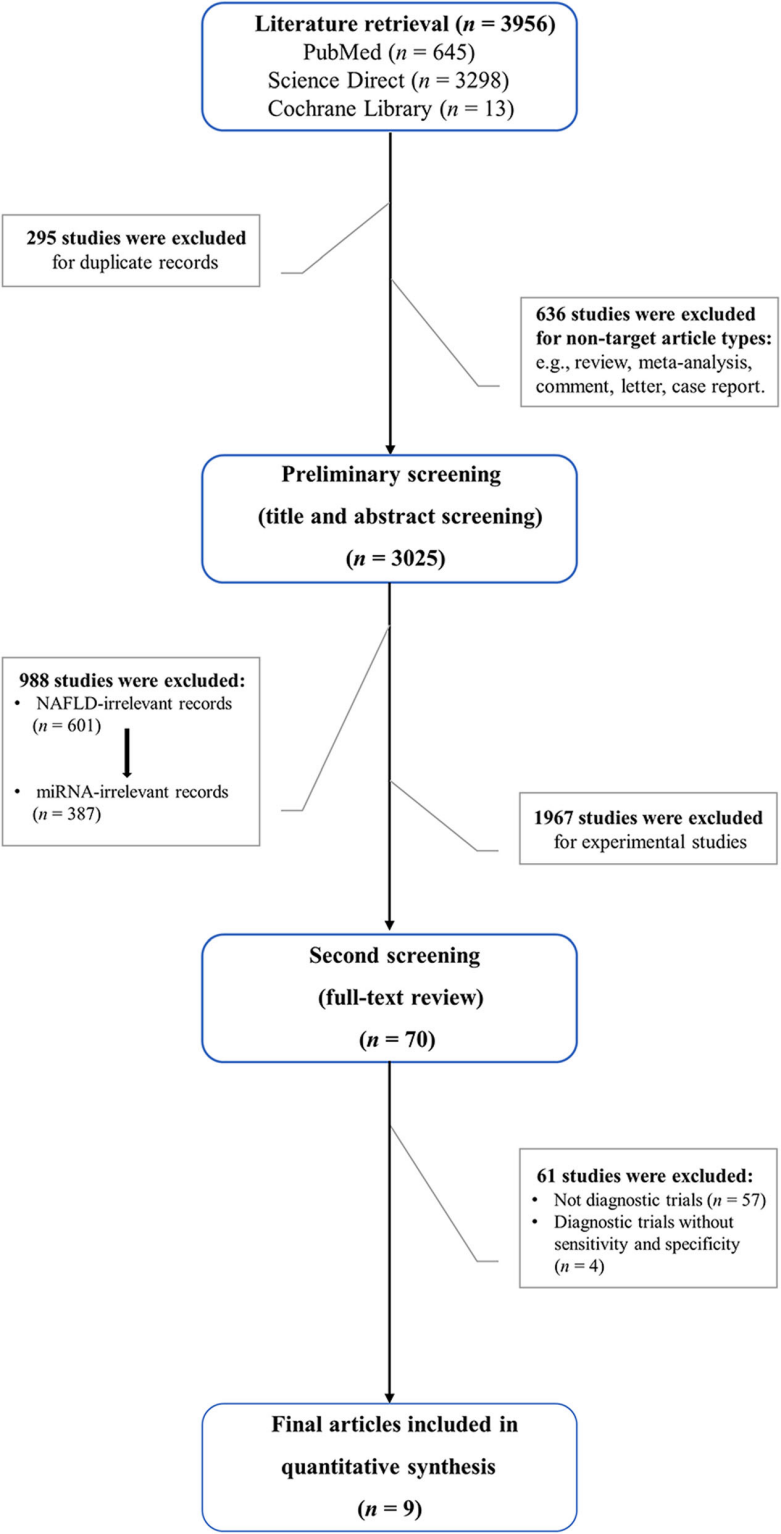
Article selection process

The literature search yielded 3956 records. Using Endnote X9, duplicate records (*n* = 295) and non-target article types (*n* = 636) were excluded. During the preliminary screening process (title and abstract screening), we eliminated irrelevant articles (*n* = 988) and experimental studies (*n* = 1967). During the second screening process (full-text review), we included nine articles that met the eligibility criteria [20–28]. A flow diagram of the literature selection process is presented in Fig. 1.

The meta-analysis was conducted on 27 trials included in nine articles. We extracted the necessary characteristics (e.g., geographic region, target miRNA, the mode of miRNA regulation, disease type, sample size, BMI, proportion of males and diagnostic sensitivity and specificity) of each trial. Trials were categorized by these factors as follows: (1) geographic region: Asian (*n* = 11), non-Asian (*n* = 16); (2) regulation mode: miRNA in upregulation mode (*n* = 21), in downregulation mode (*n* = 6); (3) disease type: NASH (*n* = 13), NAFLD (including NAFL and NASH) (*n* = 14); (4) average BMI: ≥ 30 kg/m^2^ (*n* = 15), < 30 kg/m^2^ (*n* = 9); and (5) proportion of males: ≥ 50% (*n* = 12), < 50% (*n* = 8). Detailed information of these trials is presented in Additional file 1: Table S1.

**Fig. 2:**
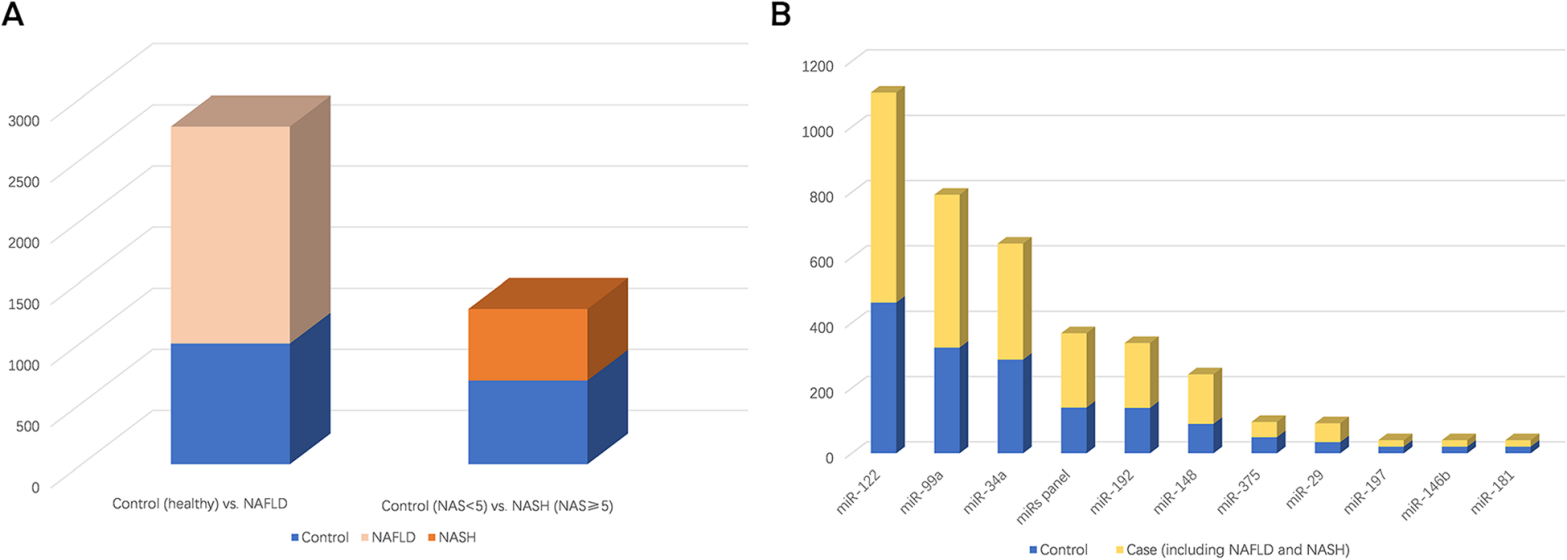
**a**: Total sample size in NAFLD trials and NASH trials. **b**: Total sample size of each target serum miRNA

The overall sample size (including controls and cases) in this meta-analysis was 4036, of which 2764 were in NAFLD trials and 1272 in NASH trials (Fig. 2a). Moreover, we summarized the target miRNAs among these 27 trials, and the top 4 in terms of total sample size were miR-122 (*n* = 1107), miRNA-99a (*n* = 792), miRNA-34a (*n* = 642) and miRNA panel (*n* = 368) (Fig. 2b). We conducted a Cochrane bias graph to assess the quality of each included article according to the QUADAS-2 tool (Additional file 1: Figure S1).

### Diagnostic value of serum miRNAs in NAFLD cases

#### Pooling all trials to evaluate the diagnostic efficacy of serum miRNA for total NAFLD

**Fig. 3:**
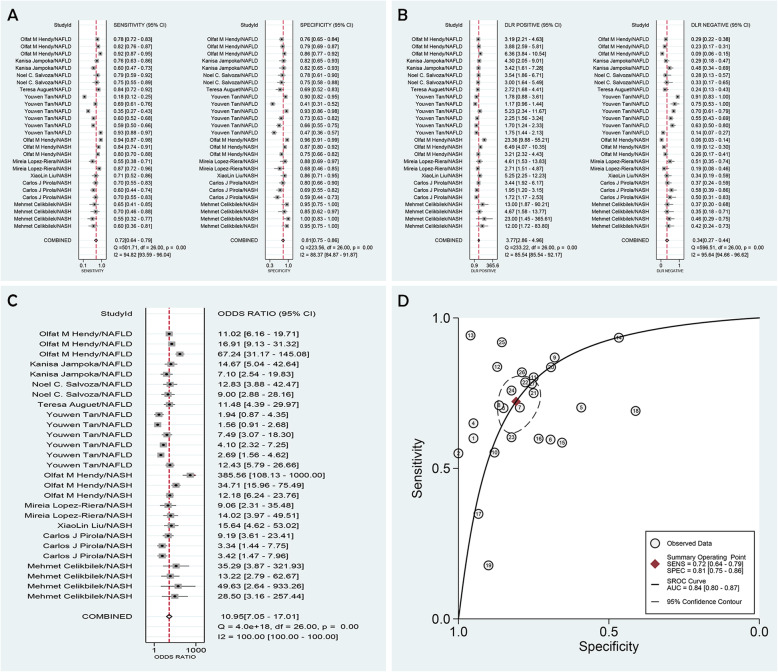
Forest plots and meta-analysis of trials showing pooled sensitivity and specificity (**a**), PLR and NLR (**b**) and DOR (**c**) of serum miRNA for diagnosis of total NAFLD (case vs. control). **d**: SROC curve of serum miRNA for diagnosis of total NAFLD (case vs. control)

Significant heterogeneity existed among the trials based on the sensitivity and specificity values (pooled *I*^*2*^ for sensitivity and specificity was 94.82 and 88.37%, respectively). Hence, we used the random-effects model in our study. The pooled values were as follows: sensitivity = 0.72 [95% confidence interval (CI): 0.64–0.79], specificity = 0.81 (95% CI: 0.75–0.86), PLR = 3.77 (95% CI: 2.86–4.96), NLR = 0.34 (95% CI: 0.27–0.44), DOR = 10.95 (95% CI: 7.05–17.01), and AUROC = 0.84 (95% CI: 0.80–0.87) (Fig. 3). These results indicated that serum miRNA had moderate diagnostic accuracy for total NAFLD.

Next, we evaluated efficacy of the most studied serum miRNAs, namely, miRNA-122, miRNA-99a and miRNA-34a, for the diagnosis of total NAFLD. (1) The pooled values of miRNA-122 were as follows: sensitivity = 0.84 (95% CI: 0.77–0.90) *I*^*2*^ = 80.62%; specificity = 0.72 (95% CI: 0.61–0.81) *I*^*2*^ = 85.44%; PLR = 3.01 (95% CI: 2.12–4.27); NLR = 0.22 (95% CI: 0.14–0.33); DOR = 13.79 (95% CI: 7.29–26.06); and AUROC = 0.86 (95% CI: 0.82–0.89) (Additional file 1: Figure S2). (2) The pooled values of miRNA-99a were as follows: sensitivity = 0.82 (95% CI: 0.71–0.89) *I*^*2*^ = 93.46%; specificity = 0.82 (95% CI: 0.53–0.95) *I*^*2*^ = 96.90%; PLR = 4.58 (95% CI: 1.30–16.12); NLR = 0.22 (95% CI: 0.11–0.47); DOR = 20.42 (95% CI: 2.86–146.00); and AUROC = 0.87 (95% CI: 0.84–0.90) (Additional file 1:Figure S3). (3) The pooled values of miRNA-34a were as follows: sensitivity = 0.81 (95% CI: 0.76–0.85) *I*^*2*^ = 5.73%; specificity = 0.83 (95% CI: 0.77–0.87) *I*^*2*^ = 33.16%; PLR = 4.70 (95% CI: 3.51–6.30); NLR = 0.23 (95% CI: 0.18–0.29); DOR = 20.34 (95% CI: 13.08–31.60); and AUROC = 0.85 (95% CI: 0.82–0.88) (Additional file 1: Figure S4). These results indicated all three miRNAs had a moderate diagnostic accuracy for total NAFLD. It should be noted that, miRNA-34a showed the lowest heterogeneity; thus, miRNA-34a was more available to diagnose total NAFLD.

#### Pooling trials NO.1–13 and trials NO.14–27 separately to evaluate the diagnostic efficacy of serum miRNA for NASH and NAFLD

1. The pooled values of NASH trials were as follows: sensitivity = 0.74 (95% CI: 0.66–0.81) *I*^*2*^ = 74.94%; specificity = 0.85 (95% CI: 0.77–0.91) *I*^*2*^ = 79.60%; PLR = 5.01 (95% CI: 3.11–8.05); NLR = 0.31 (95% CI: 0.23–0.42); DOR = 16.24 (95% CI: 8.17–32.28); and AUROC = 0.86 (95% CI: 0.83–0.89) (Additional file 1: Figure S5).
2. The pooled values of NAFLD trials were as follows: sensitivity = 0.71 (95% CI: 0.58–0.81) *I*^*2*^ = 96.88%; specificity = 0.76 (95% CI: 0.68–0.83) *I*^*2*^ = 90.57%; PLR = 2.99 (95% CI: 2.24–3.99); NLR = 0.38 (95% CI: 0.26–0.55); DOR = 7.93 (95% CI: 4.66–13.49); and AUROC = 0.80 (95% CI: 0.77–0.84) (Additional file 1: Figure S6).

These results revealed that serum miRNA had moderate diagnostic accuracy in both NASH and NAFLD trials. Moreover, serum miRNA showed a better diagnostic efficacy for NASH than that for NAFLD, as indicated by the higher DOR, higher AUROC, and lower heterogeneity.

#### Pooling trials NO.9–13 to evaluate the diagnostic efficacy of serum miRNA for distinguishing NASH from NAFL

The pooled values for these trials were as follows: sensitivity = 0.83 (95% CI: 0.70–0.91) *I*^*2*^ = 86.22%; specificity = 0.85 (95% CI: 0.74–0.92) *I*^*2*^ = 85.11%; and AUROC = 0.91 (95% CI: 0.88–0.93). These results suggested that serum miRNA had high accuracy for discriminating NASH from NAFL (Additional file 1: Figure S7).

### Subgroup analysis

To investigate the source of heterogeneity, we conducted subgroup analysis (Qualitative Research). Trials NO.1–27 were divided into subgroups based on five study factors: geographic region, type of disease, regulation mode, proportion of males and BMI. Subsequently, we calculated pooled values for each subgroup (Table 1). The results as follows.

1. *Geographic region:* compared with Asian trials, non-Asian trials had higher sensitivity (0.77 vs. 0.64) and specificity (0.83 vs. 0.76) and significantly lower heterogeneity (sensitivity *I*^*2*^ 81.33% vs. 96.08%, specificity *I*^*2*^ 76.03% vs. 92.08%). Non-Asian trials had a higher DOR (17 vs. 6) and AUROC (0.87 vs. 0.77).
2. *Type of disease:* These results were described in Section 2.2.
3. *Regulation mode:* compared with upregulation trials, downregulation trials had lower sensitivity (0.66 vs. 0.74), higher specificity (0.89 vs. 0.78), and lower heterogeneity (sensitivity *I*^*2*^ 59.82% vs. 95.92%, specificity *I*^*2*^ 58.57% vs. 90.07%). Apart from that, downregulation trials had a higher DOR (16 vs. 10) but equivalent AUROC values (0.83 vs. 0.83).
4. *Proportion of males:* compared with trials that had a proportion of males ≥50%, trials with a proportion of males < 50% had higher sensitivity (0.77 vs.0.64) and specificity (0.87 vs. 0.74) and significantly lower heterogeneity (sensitivity *I*^*2*^ 81.17% vs. 95.58%, specificity *I*^*2*^ 58.46% vs. 90.78%). Trials with a proportion of males < 50% had a higher DOR (21 vs. 5) and AUROC (0.89 vs. 0.75).
5. *BMI:* compared with trials with BMI <  30 kg/m^2^, trials with BMI ≥ 30 kg/m^2^ had higher sensitivity (0.77 vs. 0.63) and specificity (0.84 vs. 0.75) and significantly lower heterogeneity (sensitivity *I*^*2*^ 82.34% vs. 96.69%, specificity *I*^*2*^ 76.67% vs. 93.11%). Trials with BMI ≥ 30 kg/m^2^ had a higher DOR (17 vs. 5) and AUROC (0.87 vs. 0.76).

**Table 1:** Subgroup analysis for the efficacy of serum miRNA in the diagnosis of total NAFLD

Study factors	Subgroups	Case (*n*)	Sensitivity (95% CI)	*I*^*2*^ (%)	Specificity (95% CI)	*I*^*2*^ (%)	PLR (95% CI)	NLR (95% CI)	DOR (95% CI)	AUROC (95% CI)
Geographic region	Asian	11	0.64 (0.49, 0.77)	96.08	0.76 (0.65, 0.85)	92.08	2.7 (1.9, 3.7)	0.47 (0.34, 0.65)	6 (3, 9)	0.77 (0.73, 0.80)
	Non-Asian	16	0.77 (0.71, 0.83)	81.33	0.83 (0.76, 0.88)	76.03	4.5 (3.2, 6.5)	0.27 (0.20, 0.37)	17 (9, 30)	0.87 (0.84, 0.90)
Type of disease	NASH	13	0.74 (0.66, 0.81)	74.94	0.85 (0.77, 0.91)	79.60	5.0 (3.1, 8.0)	0.31 (0.23, 0.42)	16 (8, 32)	0.86 (0.83, 0.89)
	NAFLD	14	0.71 (0.58, 0.81)	96.88	0.76 (0.68, 0.83)	90.57	3.0 (2.2, 4.0)	0.38 (0.26, 0.55)	8 (5, 13)	0.80 (0.77, 0.84)
Regulation mode	Upregulation	21	0.74 (0.65, 0.81)	95.92	0.78 (0.71, 0.83)	90.07	3.4 (2.5, 4.6)	0.33 (0.24, 0.46)	10 (6, 17)	0.83 (0.80, 0.86)
	Downregulation	6	0.66 (0.56, 0.74)	59.82	0.89 (0.79, 0.95)	58.87	6.0 (3.2, 11.5)	0.38 (0.30, 0.49)	16 (8, 31)	0.83 (0.79, 0.86)
Proportion of males	≥ 50%	12	0.64 (0.50, 0.76)	95.58	0.74 (0.63, 0.82)	90.78	2.4 (1.8, 3.2)	0.49 (0.37, 0.65)	5 (3, 8)	0.75 (0.71, 0.79)
	< 50%	9	0.77 (0.66, 0.85)	81.17	0.87 (0.76, 0.93)	58.46	5.7 (3.3, 10.0)	0.27 (0.19, 0.39)	21 (11, 31)	0.89 (0.86, 0.90)
BMI	≥ 30 kg/m^2^	15	0.77 (0.69, 0.83)	82.34	0.84 (0.76, 0.89)	76.67	4.7 (3.2, 6.9)	0.28 (0.20, 0.38)	17 (9, 31)	0.87 (0.84, 0.90)
	< 30 kg/m^2^	9	0.63 (0.45, 0.78)	96.69	0.75 (0.61, 0.85)	93.11	2.5 (1.8, 3.6)	0.49 (0.34, 0.71)	5 (3, 9)	0.76 (0.72, 0.79)

*AUROC* area under the receiver operating characteristic curve, *CI* confidence interval, *DOR* diagnostic odds ratio, *NAFLD* nonalcoholic fatty liver disease, *NASH* nonalcoholic steatohepatitis, *NLR* negative likelihood ratio, *PLR* positive likelihood ratio

Collectively, serum miRNA showed more accurate diagnosis of total NAFLD in these conditions: non-Asian, presence of NASH, female predominance and BMI ≥ 30 kg/m^2^. In terms of heterogeneity, all of the above five study factors could be the potential sources.

### Meta-regression

**Fig. 4:**
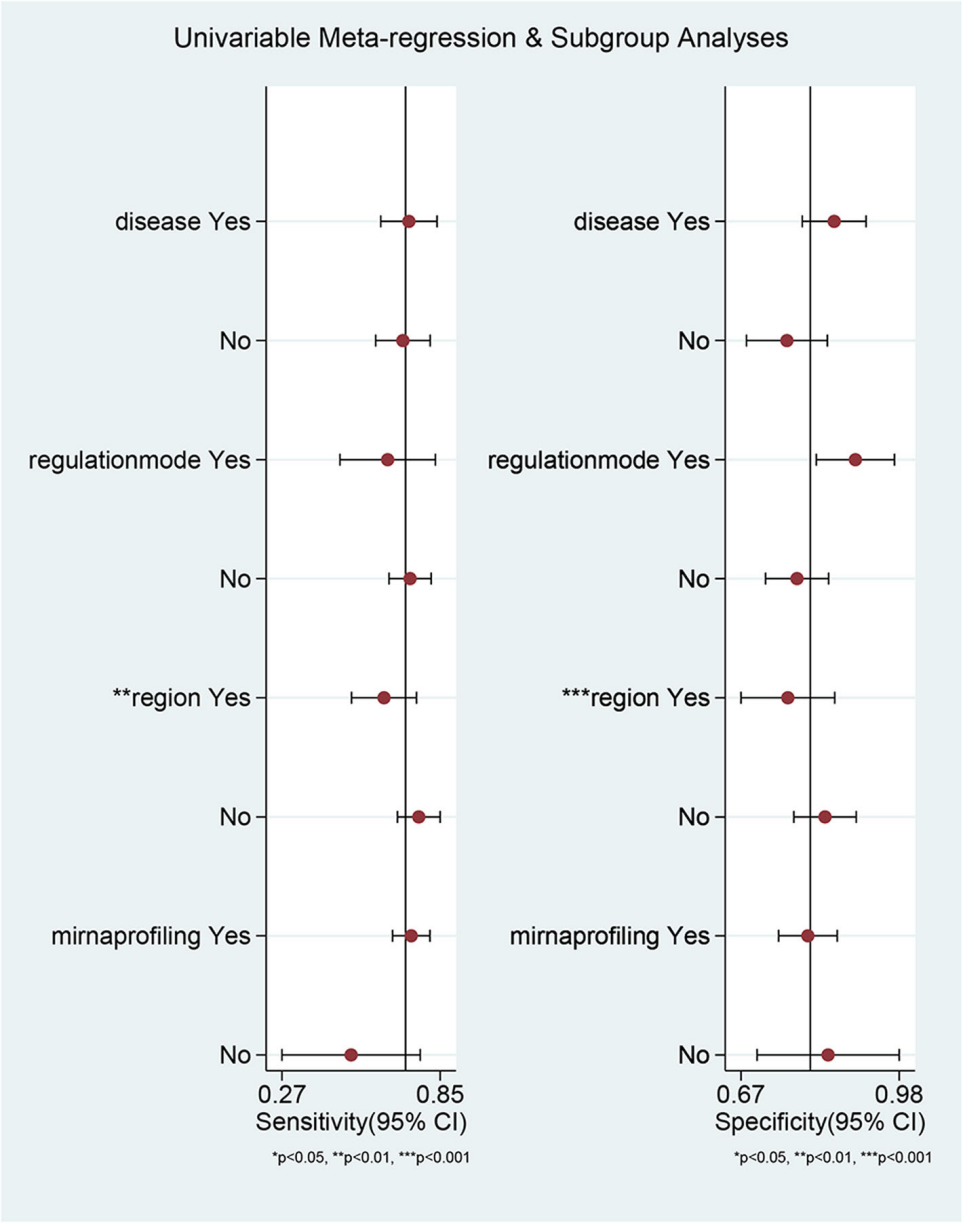
Meta-regression analysis of serum miRNA for diagnosis of total NAFLD (case vs. control). The assignment was made as follows: disease (Yes = NASH, No = NAFLD), regulation mode (Yes = upregulation, No = downregulation), geographic region (Yes = Asian, No = Non-Asian), and miRNA profiling (Yes = single miRNA, No = miRNA panel)

To determine the source of heterogeneity, we performed meta-regression (Quantitative Research). Geographic region, type of disease, miRNA regulation mode and miRNA profiling were considered as covariates. Due to lack of some data, proportion of males and BMI were not included in this meta-regression. We made the assignment as follows: disease (Yes = NASH, No = NAFLD), regulation mode (Yes = upregulation, No = downregulation), region (Yes = Asian, No = Non-Asian), and miRNA profiling (Yes = single miRNA, No = miRNA panel). The reason for heterogeneity might be related to geographic region (Asian) (sensitivity *P* < 0.01, specificity *P* < 0.001), but was unrelated to type of disease, miRNA regulation mode and miRNA profiling (Fig. 4). Thus, Asian trials could be the significant factor to induce heterogeneity.

The major difference between Asian and non-Asian trials was in BMI. Of the 16 Non-Asian trials, 15 had a BMI ≥ 30 kg/m^2^ and one did not provide the BMI. Of the 11 Asian trials, nine had a BMI <  30 kg/m^2^ and two did not provide the BMI. Moreover, the pooled values of subgroups categorized by geographic region or BMI were similar. Apart from that, no significant differences in other aspects were seen between Asian and non-Asian trials. Therefore, based on these findings, we speculated that BMI < 30 kg/m^2^ could be a potential predominant factor to induce heterogeneity rather than Asian region. After omitting trials with study factors BMI < 30 kg/m^2^, proportion of males ≥50%, NAFLD, and miRNA in upregulation mode, the heterogeneity of sensitivity and specificity showed a downward trend: sensitivity *I*^*2*^ 94.82% vs. 81.28% vs. 80.39% vs. 0%, specificity *I*^*2*^ 88.37% vs. 72.92% vs. 76.92% vs. 25.99% (Additional file 1: Table S2).

### Clinical utility

**Fig. 5:**
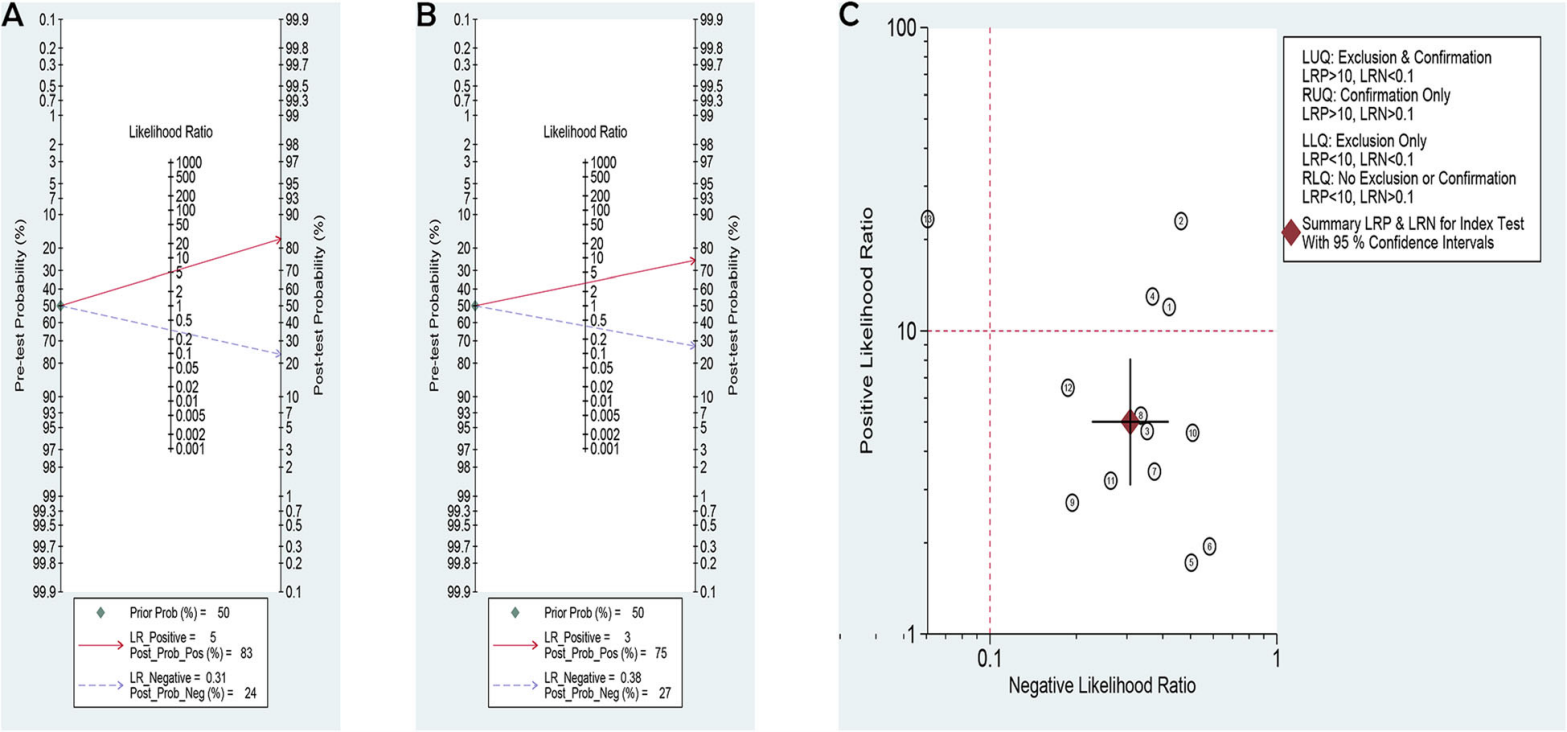
**a**: Fagan’s nomogram of serum miRNA for diagnosis of NASH (NAS ≥ 5 vs. < 5). Pre-test probability = 50% (patients with moderate suspicion for NASH), the post-test positive and negative probability of NASH were 83 and 24%, respectively. **b**: Fagan’s nomogram of serum miRNA for diagnosis of NAFLD (NAFLD vs. healthy control). Pre-test probability = 50% (patients with the moderate suspicion for NAFLD), the post-test positive and negative probability of NAFLD were 75 and 27%, respectively. **c**: The likelihood ratio scattergram of serum miRNA for diagnosis of NASH (NAS ≥ 5 vs. < 5). LRP: positive likelihood ratio; LRN: negative likelihood ratio; LUQ: left upper quadrant; RUQ: right upper quadrant; LLQ: left lower quadrant; RLQ: right lower quadrant

We used Fagan’s nomogram to examine NASH trials (NO. 1–13) and NAFLD trials (NO. 14–27 (Fig. 5a-b). Pre-test probability was set at 50%. The results were as follows. (1) Among NASH trials, the PLR was 5 accompanied by post-test probability of 83% and NLR was 0.31 accompanied by post-test probability of 24%; and (2) Among NAFLD trials, the PLR was 3 accompanied by post-test probability of 75% and NLR was 0.38 accompanied by post-test probability of 27%. These findings revealed that serum miRNA exhibited higher positive diagnostic value for NASH than that for NAFLD. Furthermore, a likelihood ratio scattergram was constructed for NASH trials (Fig. 5c). The summary likelihood ratio for serum miRNA test was located in the lower right quadrant, which meant serum miRNA test did not reach the pathological standard for exclusion and confirmation, and hence, its clinical utility was limited.

### Publication bias

**Fig. 6:**
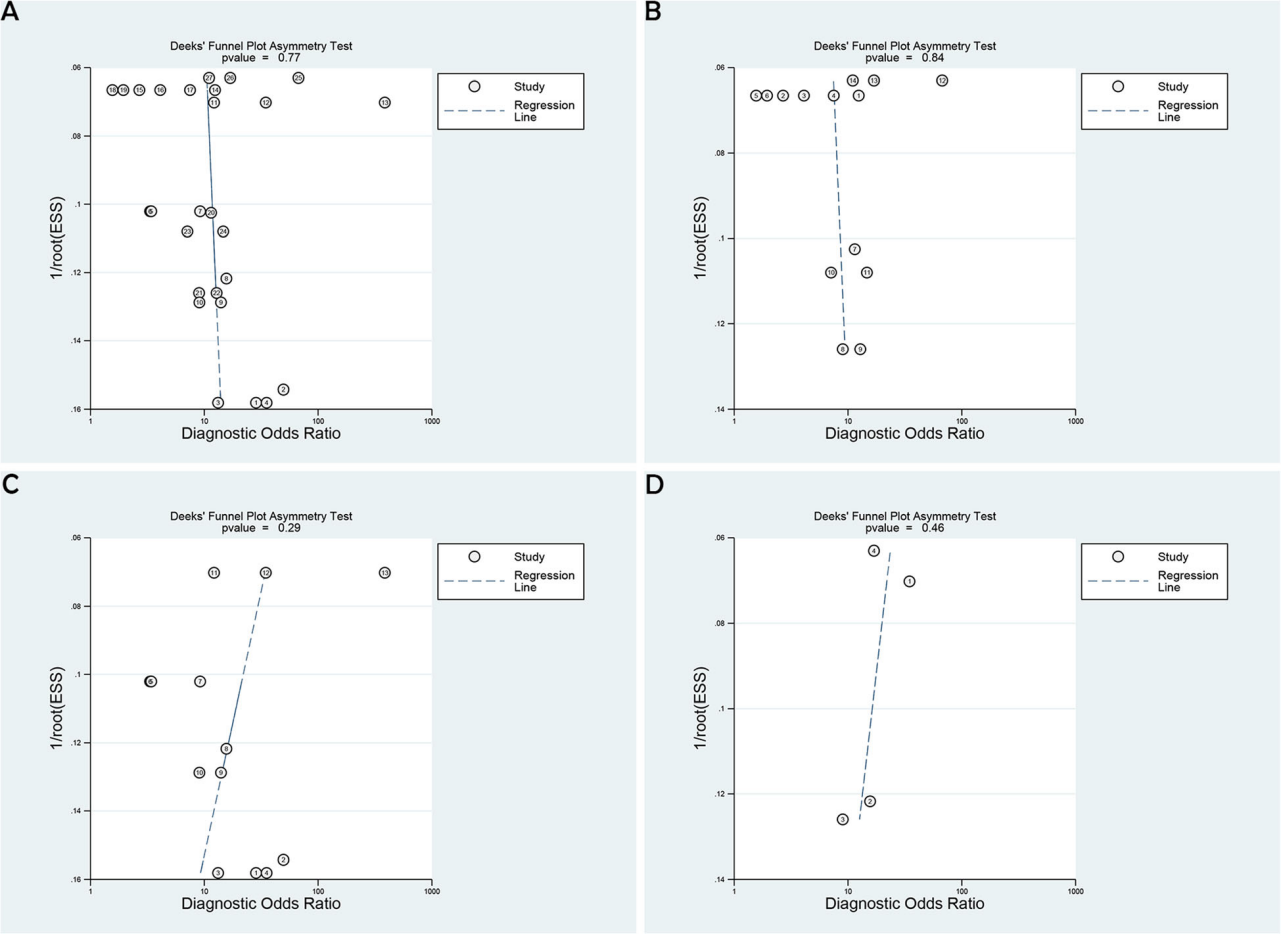
Estimation of the publication bias by Deeks’ funnel plots for all involved trials (**a**), NAFLD trials (**b**), NASH trials (**c**) and miRNA-34a trials (**d**)

Based on Deeks’ funnel plot (Fig. 6a-c), publication bias was not observed in the trials in which serum miRNA was used to diagnose total NAFLD (*P* = 0.77), NAFLD (*P* = 0.84) and NASH (*P* = 0.29). In addition, publication bias was not observed that when serum miRNA-34a was used to diagnose total NAFLD (*P* = 0.46) (Fig. 6d).

## Discussion

In this study, we systematically reviewed the diagnostic value of serum miRNA for NAFLD. Different from previous studies, we focused on NASH, which is a subtype of NAFLD. Our meta-analysis was performed on 27 trials containing 1775 NAFLD patients (including NAFL and NASH) and 586 NASH patients. We compared the diagnostic efficacy among trials from two perspectives: first, we compared the use of the most studied serum miRNAs (i.e., miRNA-122, miRNA-99 and miRNA-34a) to diagnose total NAFLD; second, we compared the use of serum miRNAs to diagnose total NAFLD, NAFLD and NASH. In addition, we detected the sources of heterogeneity by pooling data from multiple factors, including geographic region, type of disease, miRNA regulation mode, proportion of males, BMI and miRNA profiling.

### The major three conclusions of this study were as follows

(1) Serum miRNA showed a higher diagnostic efficacy for NASH than that for total NAFLD and NAFLD. Notably, serum miRNA had high accuracy in distinguishing NASH from NAFL. (2) Among the most commonly studies serum miRNAs, miRNA-34a showed the most stable efficacy, with moderate diagnostic accuracy for total NAFLD. (3) A BMI < 30 kg/m^2^ may be the predominant reason for heterogeneity. NAFLD patients with obesity (≥ 30 kg/m^2^) were more likely to have accurate diagnosis by serum miRNA test.

### First, we still need better non-invasive methods for diagnosis of NASH

NAFLD is the most common liver disease in the world. It is estimated that by 2030, the number of NAFLD patients in the United States will reach 100 million. Its subtype, NASH, accounts for 7–30% of NAFLD patients [1]. In Asia, the prevalence of NASH seems to be higher. One study found that 63.5% of liver biopsies from NAFLD patients were confirmed to be NASH [29]. NASH is an important turning point for the progression to end-stage liver disease. Therefore, the early and accurate diagnosis of NASH is needed. The gold standard for diagnosis and staging of NASH is liver biopsy. However, biopsies cannot be widely used due to complications and patients’ reluctance [30]. Therefore, non-invasive diagnostic tools are necessary. However, up to now, satisfactory biomarkers have not been identified. (1) Liver function parameters such as AST and ALT, can reflect damage of hepatocytes, but this depends on severity of NASH. Some NASH patients might present with normal levels of AST and ALT [31]. (2) CK-18 is a common index for diagnosis of NASH, because it reflects necrosis and apoptosis of hepatocytes. CK-18 is characterized by good specificity but weak sensitivity. To enhance the sensitivity of this biomarker, it needs to be combined with other indexes [7, 32–35]. (3) Inflammation indexes such as interleukin-6 and tumor necrosis factor-α, always show poor specificity [36]. (4) At present, it is reported that comprehensive scoring systems (e.g., NashTest and ActiTest) have moderate efficacy for the diagnosis of NASH, but involve excessive indexes and are costly [37]. Therefore, we still need better non-invasive methods for diagnosis of NASH.

### Second, serum miRNA has a higher diagnostic accuracy for NASH than that for NAFLD

Recent studies have revealed the close relationship between miRNA and NAFLD [38–40]. miRNAs are non-coding RNAs of 20–25 nucleotides. They have complex regulatory mechanisms. In brief, miRNAs can suppress or promote expression of target genes [41]. miRNAs widely participate in multiple pathological processes of NAFLD [14, 41], and their serum levels differ significantly between healthy individuals and NAFLD patients. Hence, they have become a new potential non-invasive biomarker for diagnosis of NAFLD. We found that serum miRNAs had moderate diagnostic accuracy for NAFLD, as reported by Cai, et al. [15]. The difference is that our subgroup analysis showed that serum miRNA has a higher diagnostic accuracy for NASH than that for NAFLD: the pooled values of NAFLD trials vs. NASH trials were sensitivity (0.71 vs. 0.74), specificity (0.76 vs. 0.85), and AUROC (0.80 vs. 0.86). It has been found that serum levels of miRNA gradually increase or decrease between healthy individuals, NAFL patients to NASH patients, which indicates disease deterioration [23, 42, 43]. That could be one reason for serum miRNAs exhibiting the better accuracy for diagnosis of NASH. Moreover, we found a high accuracy of serum miRNA for distinguishing NASH from NAFL with AUROC of 0.91. Similarly, a recent study reported that miRNA-34a had moderate accuracy for distinguishing between NASH and NAFL (AUROC = 0.78) [44]. Our study involved a total of 14 miRNAs, which have several roles in the pathogenesis of NAFLD [14, 45], such as, lipid synthesis (miRNA-122), fatty acid β-oxidation (miRNA-34a, − 122), endoplasmic reticulum stress (miRNA-30, −34a, − 122), inflammation (miRNA-34a, −99a, −146b), fibrosis (miRNA-122), tumorigenesis (miRNA-99a), and cell autophagy and apoptosis (miRNA-34a). The relationships between miRNAs and NASH pathogenesis can be both one-to-many and many-to-one. Therefore, different from traditional non-invasive diagnostic methods, serum miRNA can reflect “all-aspects” of NASH, which contributes to its diagnostic accuracy for NASH.

### Third, serum miRNA-34a is an available index to diagnose NAFLD

We focused on three well-studied miRNAs (miRNA-122, −99a, and -34a). All of them had moderate accuracy for diagnosing total NAFLD and miRNA-34a showed the lowest heterogeneity. For miRNA-122 vs. miRNA-99a vs. miRNA-34a, the pooled values were: sensitivity *I*^*2*^ (80.62% vs. 93.46% vs. 5.73%), specificity *I*^*2*^ (85.44% vs. 96.90% vs. 33.16%), and AUROC (0.86 vs. 0.87 vs. 0.85). miRNA-34a exhibited the most stable efficacy among the three miRNAs. Therefore, serum miRNA-34a is a more available biomarker to diagnose NAFLD. Previous studies have shown that miRNA-34a plays pivotal roles in NAFLD progression. (1) In terms of lipid metabolism, miRNA-34a can downregulate the PPARα signaling pathway. PPARα is a key transcription factor of fatty acid oxidation that facilitates the transfer of fatty acids into the mitochondria for oxidation. On the contrary, blocking the PPARα signaling pathway could induce lipid accumulation in hepatocytes [46]. (2) In addition, PPARα is involved in liver inflammation by activating Kupffer cells [47]. (3) In terms of apoptosis, by repressing sirtuin-1, miRNA-34a increases p53 acetylation and transcription, leading to induction of pro-apoptotic genes such as *PUMA*, and finally, apoptosis [48]. Collectively, miRNA-34a moderates the “first hit” (abnormal lipid metabolism) and “second hit” (inflammation and apoptosis) for the pathogenesis of NAFLD. In other words, miRNA-34a could reflect the entire course of NAFLD, including onset and subsequent progression. Therefore, that is one reason for miRNA-34a exhibiting more stable diagnostic efficacy than other miRNAs. We did not evaluate the diagnostic efficacy of miRNA-34a for NASH separately. A previous study has found that miRNA-34a can distinguish NASH from NAFL with an AUROC of 0.78 [44].

### Fourth, the diagnostic efficacy of serum miRNA for NAFLD may depend on BMI

In subgroup analysis, miRNA showed greater accuracy for diagnosing NAFLD in patients with BMI ≥ 30 kg/m^2^. The pooled values of trials with BMI ≥ 30 kg/m^2^ and < 30 kg/m^2^ were: sensitivity (0.77 vs.0.63), specificity (0.84 vs. 0.75), and AUROC (0.87 vs. 0.76). In meta-regression, the covariate, geographic region (Asian), demonstrated significant differences in sensitivity and specificity. Thus, Asian region could be a significant factor for heterogeneity. Moreover, in view of the obvious overlap when trials were separately categorized by geographic region and BMI, we speculated that BMI < 30 kg/m^2^ was the potential predominant factor for heterogeneity rather than Asian region. When we omitted the trials with BMI < 30 kg/m^2^, heterogeneity of sensitivity and specificity were mitigated: sensitivity *I*^*2*^ 94.82% vs. 81.28%, specificity *I*^*2*^ 88.37% vs. 72.92%. In our study, Asian patients with NAFLD were characterized by lower BMIs, which ranged from 24 to 28 kg/m^2^. A recent epidemiological investigation supports our findings: the prevalence of NAFLD in people with BMI < 25 kg/m^2^ was 8–20% in China, 7% in India, 15% in Korea and 13% in Japan. Lean or non-obese NAFLD has become a new trend in Asia [49]. The pathogenesis of lean NAFLD and obese NAFLD are not entirely consistent. Studies have found that *PNPLA3* polymorphism plays a specific role in NAFLD in the non-obese population [50, 51]. Considering this peculiarity, we do not recommend the application of serum miRNA test for diagnosis in NAFLD patients with BMI < 30 kg/m^2^. Collectively, the diagnostic efficacy of serum miRNA for NAFLD may depend on BMI. Due to lack of some data, we did not directly evaluate the effect of BMI on heterogeneity by meta-regression. Thus, it needs to be confirmed in a further study.

### Fifth, the serum miRNA test could be used for the early diagnosis of NASH in clinical practice

(1) In general, serum miRNA showed moderate efficacy for diagnosis of NASH. In terms of clinical utility, Fagan’s nomogram revealed that when a patient was in a dilemma about diagnosis or exclusion of NASH, miRNA could provide an effective reference: if a positive result was acquired, the patient would have 83% probability of confirmation by liver biopsy; If a negative result was acquired, the patient would has 76% probability of exclusion by liver biopsy. (2) Individually, because of the necessity for early diagnosis of NASH in clinical practice, sensitivity deserves special attention. Our study suggests that serum miRNA shows robust positive efficacy for diagnosis of NASH: NASH trials had higher pooled sensitivity (0.74 vs. 0.71), PLR (5.01 vs. 2.99), DOR(16.24 vs. 7.93), and post-test positive probability (83% vs. 75%) than those of NAFLD trials; pooled sensitivity was further increased to 0.83 when distinguishing NASH from NAFL; likelihood ratio scattergram indicated that more trials (*n* = 4 vs. 1) reached the pathological confirmation standard (positive likelihood ratio, LRP > 10) rather than pathological exclusion standard (negative likelihood ratio, LRN < 0.1). (3) The serum miRNA test has a limited clinical utility. In the likelihood ratio scattergram, the summary LRP & LRN for serum miRNA test was located in the right lower quadrant, which means that serum miRNA test still has less in clinical utility than the gold standard, liver biopsy. Therefore, we should improve this test in the future as follows: the diagnostic flow of serum miRNA should be standardized, including sampling and testing; dynamic monitoring should be performed; this method should be applied mostly among obese individuals (BMI ≥ 30 kg/m^2^); this method should be combined with other non-invasive methods, such as CK-18, which has a good specificity but a weak sensitivity, to enhance the overall diagnostic efficacy. In summary, there are still few accurate and non-invasive diagnostic methods that can replace liver biopsy. As a novel and effective method, the serum miRNA test is quantitative in real-time and easy to conduct. When suspected NASH patients present with normal liver function parameters, an early serum miRNA test should be considered.

### Last, there are still some limitations to this study

First, the cutoff value was an important cause of heterogeneity; however, most trials in this study did not provide a cutoff value. Second, we extracted multiple trials from one article, which may have increased statistical deviation. Third, due to limited numbers of trials, we did not evaluate the efficacy of miRNA-34a for diagnosis of NASH. Fourth, due to a lack of some data, we did not directly analyze the BMI by meta-regression; Finally, we may have ignored some available literature.

## Conclusion

Based on the above analysis, we conclude that serum miRNA could be considered as a promising non-invasive test for diagnosis of NASH. In particular, serum miRNA may provide effective guidance to clinicians on early diagnosis because of its prominent sensitivity. Moreover, among the well-studied serum miRNAs, miRNA-34a is the most available diagnostic index for NAFLD. It should be noticed that BMI may influence the diagnostic performance, and more research on this aspect is necessary in the future.

## Supplementary information

**Additional file 1: Figure S1.** Quality assessment of the included trials. **Figure S2.** Diagnostic efficacy of serum miRNA-122 for total NAFLD (case vs. control). **Figure S3.** Diagnostic efficacy of serum miRNA-99a for total NAFLD (case vs. control). **Figure S4.** Diagnostic efficacy of serum miRNA-34a for total NAFLD (case vs. control). **Figure S5.** Diagnostic efficacy of serum miRNA for NASH (NAS ≥ 5 vs. < 5). **Figure S6.** Diagnostic efficacy of serum miRNA for NAFLD (NAFLD vs. healthy control). **Figure S7.** Diagnostic efficacy of serum miRNA for distinguishing NASH from NAFL. **Table S1.** Basic characteristics of the included trials. **Table S2.** Heterogeneity of serum miRNA for diagnosing total NAFLD after omitting the trials with study factors. PRISMA 2009 Checklist.

## Data Availability

The datasets used and/or analyzed during the current study are available from the corresponding authors upon reasonable request.

## Abbreviations

ALT: Alanine aminotransferase
AST: Aspartate aminotransferase
AUROC: Area under the receiver operating characteristics curve
BMI: Body mass index
CI: Confidence interval
CK-18: Cytokeratin 18
DOR: Diagnostic odds ratio
FN: False negative
FP: False positive
PLR: Positive likelihood ratio
NAFL: Nonalcoholic fatty liver (simple steatosis)
NAFLD: Nonalcoholic fatty liver disease
NAS: NAFLD activity score
NASH: Nonalcoholic steatohepatitis
NLR: Negative likelihood ratio
PPARα: Peroxisome proliferator-activated receptor α
PNPLA3: Patatin like phospholipase domain containing 3
PUMA: p53 up-regulated modulator of apoptosis
QUADAS: Quality assessment of diagnostic accuracy studies
RT-PCR: Reverse transcription-polymerase chain reaction
SROC: Summary receiver-operating characteristics
TN: True negative
TP: True positive
