# Supplementary material for: Efficacy of serum miRNA test as a non-invasive method to diagnose nonalcoholic steatohepatitis: a systematic review and meta-analysis
Source: BMC Gastroenterol. 2020 Jun 12;20:186. doi: 10.1186/s12876-020-01334-8 (PMC7291448; doi:10.1186/s12876-020-01334-8)
Supplement: Supplementary file 1 — Additional file 1: Figure S1. Quality assessment of the included trials. Figure S2. Diagnostic efficacy of serum miRNA-122 for total NAFLD (case vs. control). Figure S3. Diagnostic efficacy of serum miRNA-99a for total NAFLD (case vs. control). Figure S4. Diagnostic efficacy of serum miRNA-34a for total NAFLD (case vs. control). Figure S5. Diagnostic efficacy of serum miRNA for NASH (NAS ≥ 5 vs. < 5). Figure S6. Diagnostic efficacy of serum miRNA for NAFLD (NAFLD vs. healthy control). Figure S7. Diagnostic efficacy of serum miRNA for distinguishing NASH from NAFL. Table S1. Basic characteristics of the included trials. Table S2. Heterogeneity of serum miRNA for diagnosing total NAFLD after omitting the trials with study factors. PRISMA 2009 Checklist. [file 12876_2020_1334_MOESM1_ESM.pdf]

Efficacy of serum miRNA test as a non-invasive method to diagnose nonalcoholic steatohepatitis: a systematic review and meta-analysis.

Shengliang Xin[1], Qiao Zhan[1], Xiaofan Chen[1], Jinghang Xu[1], Yanyan Yu[1]

Contents of Supplementary Information

Supplementary figures

Figure S1

Quality assessment of the included trials.

A: methodological quality graph; B: Cochrane Handbook.

Figure S2

Diagnostic efficacy of serum miRNA-122 for total NAFLD (case vs. control).

Forest plots and meta-analysis of trials showing pooled sensitivity and specificity (A), PLR and NLR (B) and DOR (C) of serum miRNA-122 for diagnosis of total NAFLD (case vs. control). D: SROC curve of serum miRNA-122 for diagnosis of total NAFLD (case vs. control).

Figure S3

Diagnostic efficacy of serum miRNA-99a for total NAFLD (case vs. control).

Forest plots and meta-analysis of trials showing pooled sensitivity and specificity (A), PLR and NLR (B) and DOR (C) of serum miRNA-99a for diagnosis of total NAFLD (case vs. control). D: SROC curve of serum miRNA-99a for diagnosis of total NAFLD (case vs. control).

Figure S4

Diagnostic efficacy of serum miRNA-34a for total NAFLD (case vs. control).

Forest plots and meta-analysis of trials showing pooled sensitivity and specificity (A), PLR and NLR (B) and DOR (C) of serum miRNA-34a for diagnosis of total NAFLD (case vs. control). D:

SROC curve of serum miRNA-34a for diagnosis of total NAFLD (case vs. control).

Figure S5

Diagnostic efficacy of serum miRNA for NASH (NAS ≥ 5 vs. < 5).

Forest plots and meta-analysis of trials showing pooled sensitivity and specificity (A), PLR and NLR (B) and DOR (C) of serum miRNA for diagnosis of NASH (NAS ≥ 5 vs. < 5). D: SROC curve of serum miRNA for diagnosis of NASH (NAS ≥ 5 vs. < 5).

Figure S6

Diagnostic efficacy of serum miRNA for NAFLD (NAFLD vs. healthy control).

Forest plots and meta-analysis of trials showing pooled sensitivity and specificity (A), PLR and NLR (B) and DOR (C) of serum miRNA for diagnosis of NAFLD (NAFLD vs. healthy control). D: SROC curve of serum miRNA for diagnosis of NAFLD (NAFLD vs. healthy control).

Figure S7

Diagnostic efficacy of serum miRNA for distinguishing NASH from NAFL.

Forest plots and meta-analysis of trials showing pooled sensitivity and specificity (A) for distinguishing NASH from NAFL. D: SROC curve of serum miRNA for distinguishing NASH from NAFL.

Supplementary tables

Table S1.

Basic characteristics of the included trials.

Table S2.

Heterogeneity of serum miRNA for diagnosing total NAFLD after omitting the trials with study

factors.

PRISMA 2009 Checklist

Figure S1

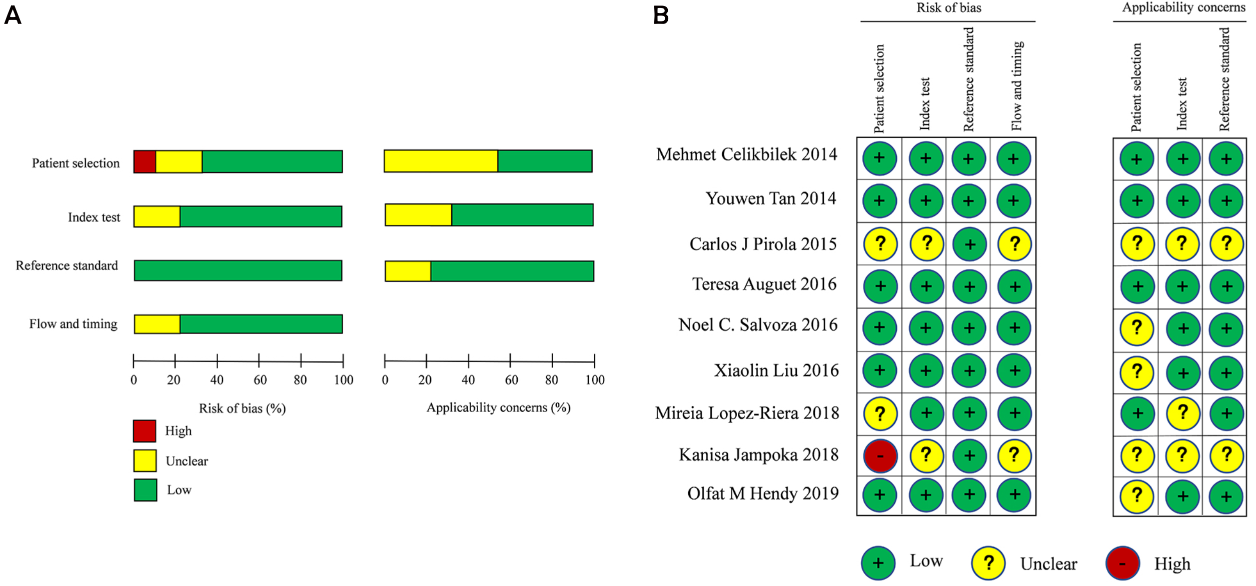
A
Patient selection
Index test
Reference standard
Flow and timing
0
20
40
60
80
100
Risk of bias (%)
Applicability concerns (%)
High
Unclear
Low
B
Risk of bias
Applicability concerns
Patient selection
Index test
Reference standard
Flow and timing
Mehmet Celikbilek 2014
Youwen Tan 2014
Carlos J Pirola 2015
Teresa Auguet 2016
Noel C. Salvoza 2016
Xiaolin Liu 2016
Mireia Lopez-Riera 2018
Kanisa Jampoka 2018
Olfat M Hendy 2019
Low
Unclear
High

Figure S2

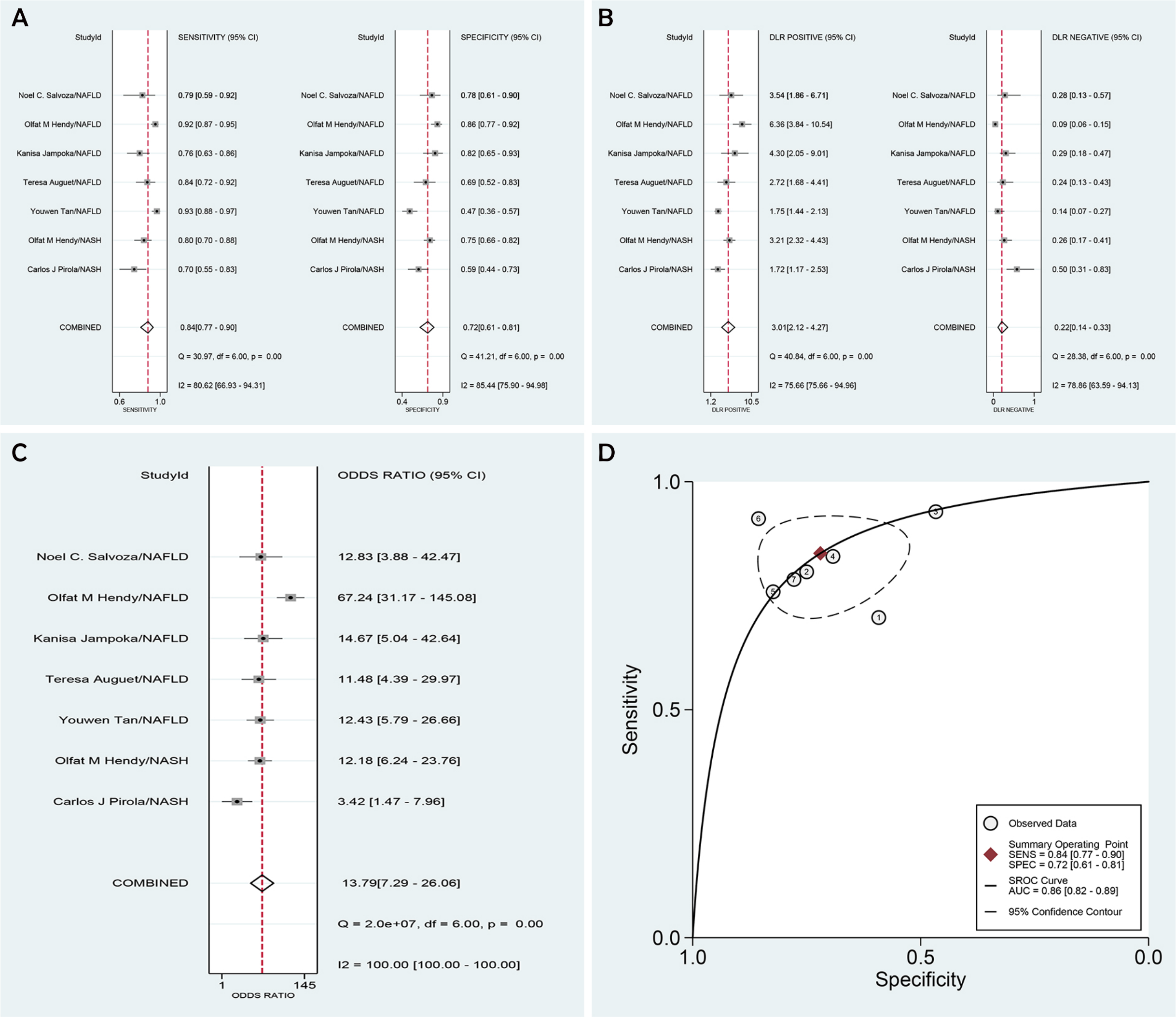

A
StudyId
SENSITIVITY (95% CI)
Noel C. Salvoza/NAFLD 0.79 [0.59 - 0.92]
Olfat M Hendy/NAFLD 0.92 [0.87 - 0.95]
Kanisa Jampoka/NAFLD 0.76 [0.63 - 0.86]
Teresa Auguet/NAFLD 0.84 [0.72 - 0.92]
Youwen Tan/NAFLD 0.93 [0.88 - 0.97]
Olfat M Hendy/NASH 0.80 [0.70 - 0.88]
Carlos J Pirola/NASH 0.70 [0.55 - 0.83]
COMBINED 0.84[0.77 - 0.90]
Q = 30.97, df = 6.00, p = 0.00
I2 = 80.62 [66.93 - 94.31]
0.6 1.0
SENSITIVITY
StudyId
SPECIFICITY (95% CI)
Noel C. Salvoza/NAFLD 0.78 [0.61 - 0.90]
Olfat M Hendy/NAFLD 0.86 [0.77 - 0.92]
Kanisa Jampoka/NAFLD 0.82 [0.65 - 0.93]
Teresa Auguet/NAFLD 0.69 [0.52 - 0.83]
Youwen Tan/NAFLD 0.47 [0.36 - 0.57]
Olfat M Hendy/NASH 0.75 [0.66 - 0.82]
Carlos J Pirola/NASH 0.59 [0.44 - 0.73]
COMBINED 0.72[0.61 - 0.81]
Q = 41.21, df = 6.00, p = 0.00
I2 = 85.44 [75.90 - 94.98]
0.4 0.9
SPECIFICITY
B
StudyId
DLR POSITIVE (95% CI)
Noel C. Salvoza/NAFLD 3.54 [1.86 - 6.71]
Olfat M Hendy/NAFLD 6.36 [3.84 - 10.54]
Kanisa Jampoka/NAFLD 4.30 [2.05 - 9.01]
Teresa Auguet/NAFLD 2.72 [1.68 - 4.41]
Youwen Tan/NAFLD 1.75 [1.44 - 2.13]
Olfat M Hendy/NASH 3.21 [2.32 - 4.43]
Carlos J Pirola/NASH 1.72 [1.17 - 2.53]
COMBINED 3.01[2.12 - 4.27]
Q = 40.84, df = 6.00, p = 0.00
I2 = 75.66 [75.66 - 94.96]
1.2 10.5
DLR POSITIVE
StudyId
DLR NEGATIVE (95% CI)
Noel C. Salvoza/NAFLD 0.28 [0.13 - 0.57]
Olfat M Hendy/NAFLD 0.09 [0.06 - 0.15]
Kanisa Jampoka/NAFLD 0.29 [0.18 - 0.47]
Teresa Auguet/NAFLD 0.24 [0.13 - 0.43]
Youwen Tan/NAFLD 0.14 [0.07 - 0.27]
Olfat M Hendy/NASH 0.26 [0.17 - 0.41]
Carlos J Pirola/NASH 0.50 [0.31 - 0.83]
COMBINED 0.22[0.14 - 0.33]
Q = 28.38, df = 6.00, p = 0.00
I2 = 78.86 [63.59 - 94.13]
0 1
DLR NEGATIVE
C
StudyId
ODDS RATIO (95% CI)
Noel C. Salvoza/NAFLD 12.83 [3.88 - 42.47]
Olfat M Hendy/NAFLD 67.24 [31.17 - 145.08]
Kanisa Jampoka/NAFLD 14.67 [5.04 - 42.64]
Teresa Auguet/NAFLD 11.48 [4.39 - 29.97]
Youwen Tan/NAFLD 12.43 [5.79 - 26.66]
Olfat M Hendy/NASH 12.18 [6.24 - 23.76]
Carlos J Pirola/NASH 3.42 [1.47 - 7.96]
COMBINED 13.79[7.29 - 26.06]
Q = 2.0e+07, df = 6.00, p = 0.00
I2 = 100.00 [100.00 - 100.00]
1 145
ODDS RATIO
D
1.0
0.5
0.0
Sensitivity
1.0
0.5
0.0
Specificity
Observed Data
Summary Operating Point
SENS = 0.84 [0.77 - 0.90]
SPEC = 0.72 [0.61 - 0.81]
SROC Curve
AUC = 0.86 [0.82 - 0.89]
95% Confidence Contour

Figure S3

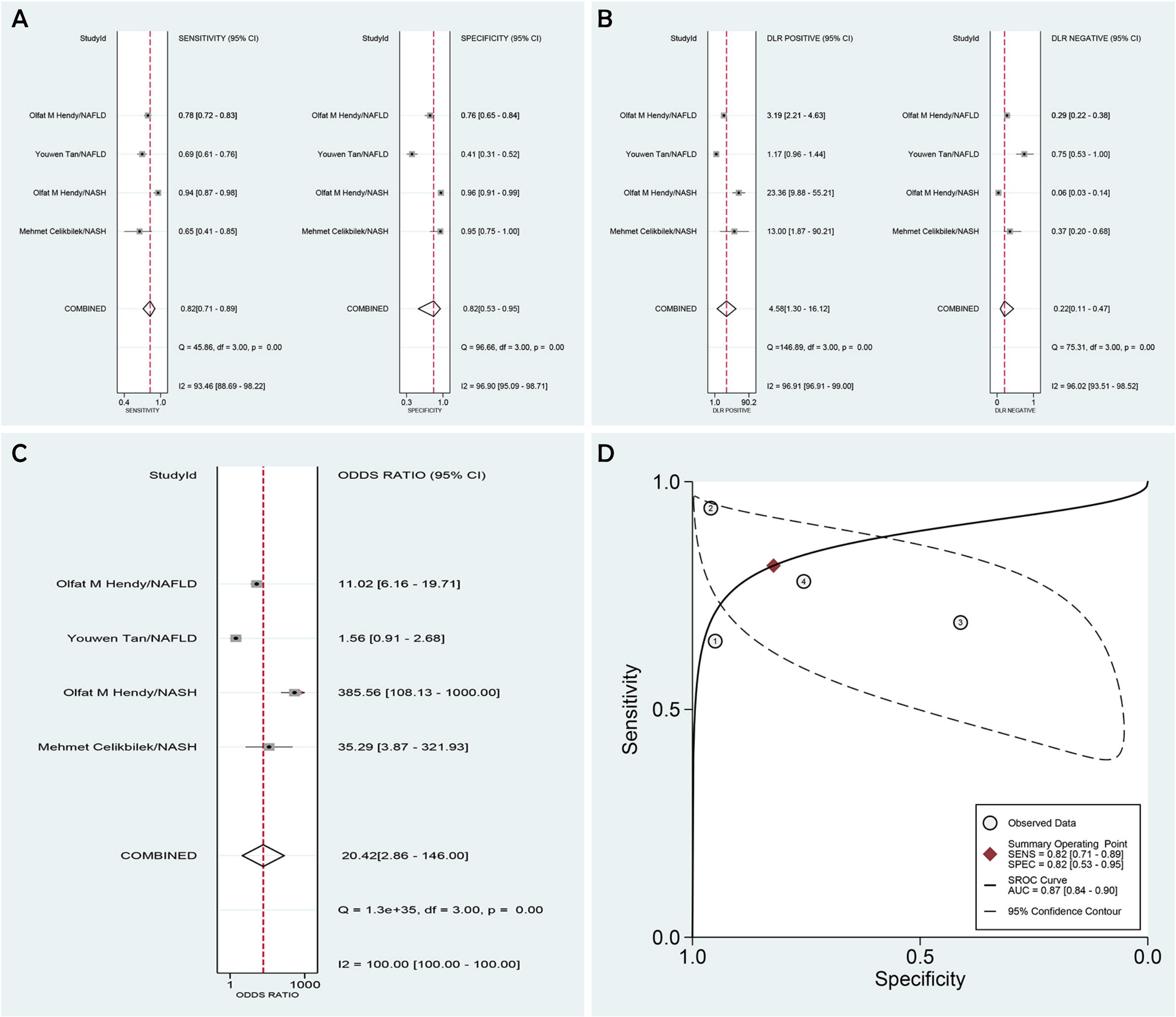
A
StudyId
SENSITIVITY (95% CI)
Olfat M Hendy/NAFLD
0.78 [0.72 - 0.83]
Youwen Tan/NAFLD
0.69 [0.61 - 0.76]
Olfat M Hendy/NASH
0.94 [0.87 - 0.98]
Mehmet Celikbilek/NASH
0.65 [0.41 - 0.85]
COMBINED
0.82[0.71 - 0.89]
Q = 45.86, df = 3.00, p = 0.00
I2 = 93.46 [88.69 - 98.22]
0.4
1.0
SENSITIVITY
StudyId
SPECIFICITY (95% CI)
Olfat M Hendy/NAFLD
0.76 [0.65 - 0.84]
Youwen Tan/NAFLD
0.41 [0.31 - 0.52]
Olfat M Hendy/NASH
0.96 [0.91 - 0.99]
Mehmet Celikbilek/NASH
0.95 [0.75 - 1.00]
COMBINED
0.82[0.53 - 0.95]
Q = 96.66, df = 3.00, p = 0.00
I2 = 96.90 [95.09 - 98.71]
0.3
1.0
SPECIFICITY
B
StudyId
DLR POSITIVE (95% CI)
Olfat M Hendy/NAFLD
3.19 [2.21 - 4.63]
Youwen Tan/NAFLD
1.17 [0.96 - 1.44]
Olfat M Hendy/NASH
23.36 [9.88 - 55.21]
Mehmet Celikbilek/NASH
13.00 [1.87 - 90.21]
COMBINED
4.58[1.30 - 16.12]
Q =146.89, df = 3.00, p = 0.00
I2 = 96.91 [96.91 - 99.00]
1.0
90.2
DLR POSITIVE
StudyId
DLR NEGATIVE (95% CI)
Olfat M Hendy/NAFLD
0.29 [0.22 - 0.38]
Youwen Tan/NAFLD
0.75 [0.53 - 1.00]
Olfat M Hendy/NASH
0.06 [0.03 - 0.14]
Mehmet Celikbilek/NASH
0.37 [0.20 - 0.68]
COMBINED
0.22[0.11 - 0.47]
Q = 75.31, df = 3.00, p = 0.00
I2 = 96.02 [93.51 - 98.52]
0
1
DLR NEGATIVE
C
StudyId
ODDS RATIO (95% CI)
Olfat M Hendy/NAFLD
11.02 [6.16 - 19.71]
Youwen Tan/NAFLD
1.56 [0.91 - 2.68]
Olfat M Hendy/NASH
385.56 [108.13 - 1000.00]
Mehmet Celikbilek/NASH
35.29 [3.87 - 321.93]
COMBINED
20.42[2.86 - 146.00]
Q = 1.3e+35, df = 3.00, p = 0.00
I2 = 100.00 [100.00 - 100.00]
1
1000
ODDS RATIO
D
1.0
0.5
0.0
Sensitivity
1.0
0.5
0.0
Specificity
1
2
3
4
Observed Data
Summary Operating Point
SENS = 0.82 [0.71 - 0.89]
SPEC = 0.82 [0.53 - 0.95]
SROC Curve
AUC = 0.87 [0.84 - 0.90]
95% Confidence Contour

Figure S4

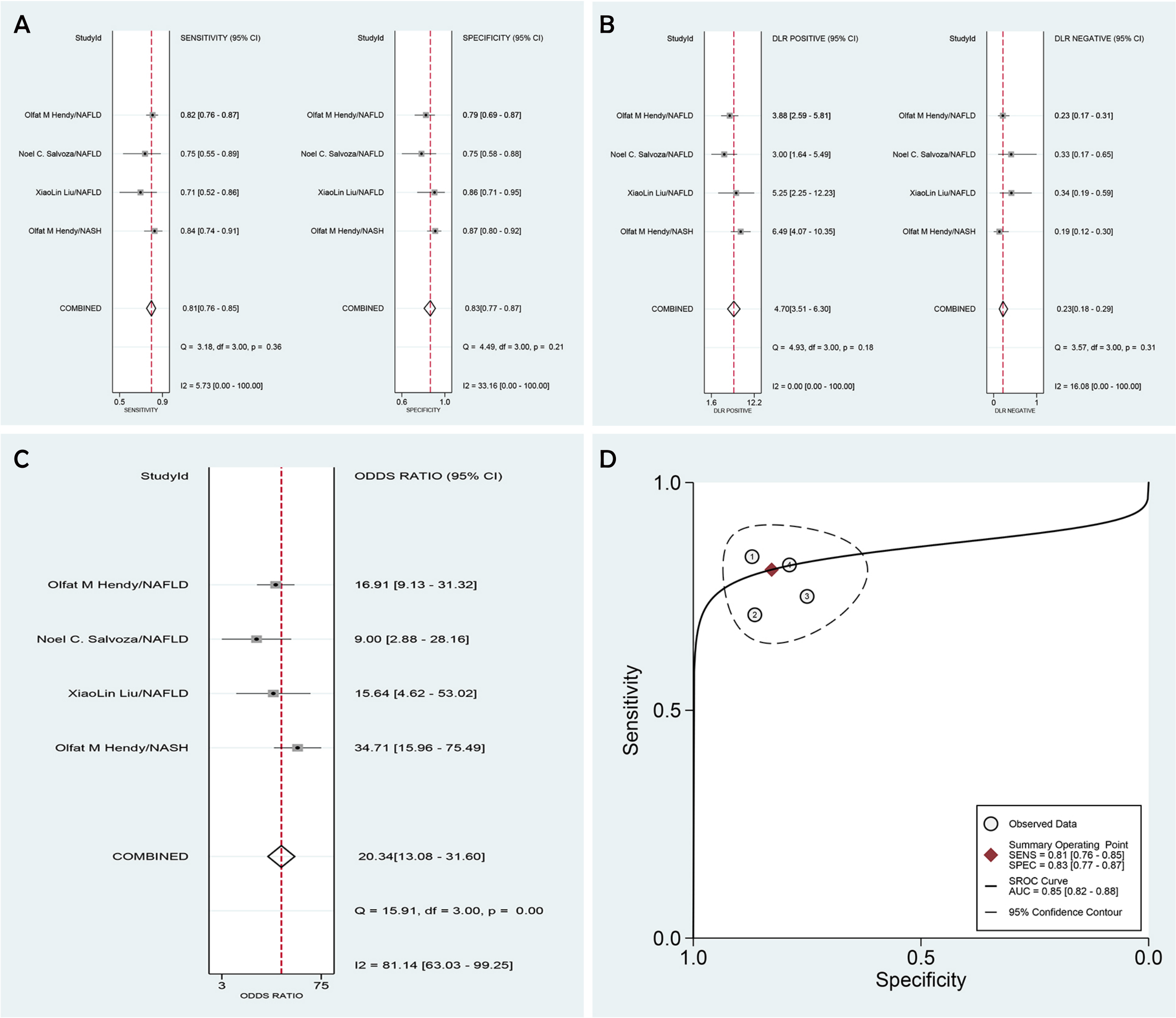

A
StudyId
SENSITIVITY (95% CI)
Olfat M Hendy/NAFLD 0.82 [0.76 - 0.87]
Noel C. Salvoza/NAFLD 0.75 [0.55 - 0.89]
XiaoLin Liu/NAFLD 0.71 [0.52 - 0.86]
Olfat M Hendy/NASH 0.84 [0.74 - 0.91]
COMBINED 0.81[0.76 - 0.85]
Q = 3.18, df = 3.00, p = 0.36
I2 = 5.73 [0.00 - 100.00]
0.5 0.9
SENSITIVITY
StudyId
SPECIFICITY (95% CI)
Olfat M Hendy/NAFLD 0.79 [0.69 - 0.87]
Noel C. Salvoza/NAFLD 0.75 [0.58 - 0.88]
XiaoLin Liu/NAFLD 0.86 [0.71 - 0.95]
Olfat M Hendy/NASH 0.87 [0.80 - 0.92]
COMBINED 0.83[0.77 - 0.87]
Q = 4.49, df = 3.00, p = 0.21
I2 = 33.16 [0.00 - 100.00]
0.6 1.0
SPECIFICITY
B
StudyId
DLR POSITIVE (95% CI)
Olfat M Hendy/NAFLD 3.88 [2.59 - 5.81]
Noel C. Salvoza/NAFLD 3.00 [1.64 - 5.49]
XiaoLin Liu/NAFLD 5.25 [2.25 - 12.23]
Olfat M Hendy/NASH 6.49 [4.07 - 10.35]
COMBINED 4.70[3.51 - 6.30]
Q = 4.93, df = 3.00, p = 0.18
I2 = 0.00 [0.00 - 100.00]
1.6 12.2
DLR POSITIVE
StudyId
DLR NEGATIVE (95% CI)
Olfat M Hendy/NAFLD 0.23 [0.17 - 0.31]
Noel C. Salvoza/NAFLD 0.33 [0.17 - 0.65]
XiaoLin Liu/NAFLD 0.34 [0.19 - 0.59]
Olfat M Hendy/NASH 0.19 [0.12 - 0.30]
COMBINED 0.23[0.18 - 0.29]
Q = 3.57, df = 3.00, p = 0.31
I2 = 16.08 [0.00 - 100.00]
0 1
DLR NEGATIVE
C
StudyId
ODDS RATIO (95% CI)
Olfat M Hendy/NAFLD 16.91 [9.13 - 31.32]
Noel C. Salvoza/NAFLD 9.00 [2.88 - 28.16]
XiaoLin Liu/NAFLD 15.64 [4.62 - 53.02]
Olfat M Hendy/NASH 34.71 [15.96 - 75.49]
COMBINED 20.34[13.08 - 31.60]
Q = 15.91, df = 3.00, p = 0.00
I2 = 81.14 [63.03 - 99.25]
3 75
ODDS RATIO
D
1.0
0.5
0.0
Sensitivity
1.0 0.5 0.0
Specificity
Observed Data
Summary Operating Point
SENS = 0.81 [0.76 - 0.85]
SPEC = 0.83 [0.77 - 0.87]
SROC Curve
AUC = 0.85 [0.82 - 0.88]
95% Confidence Contour

Figure S5

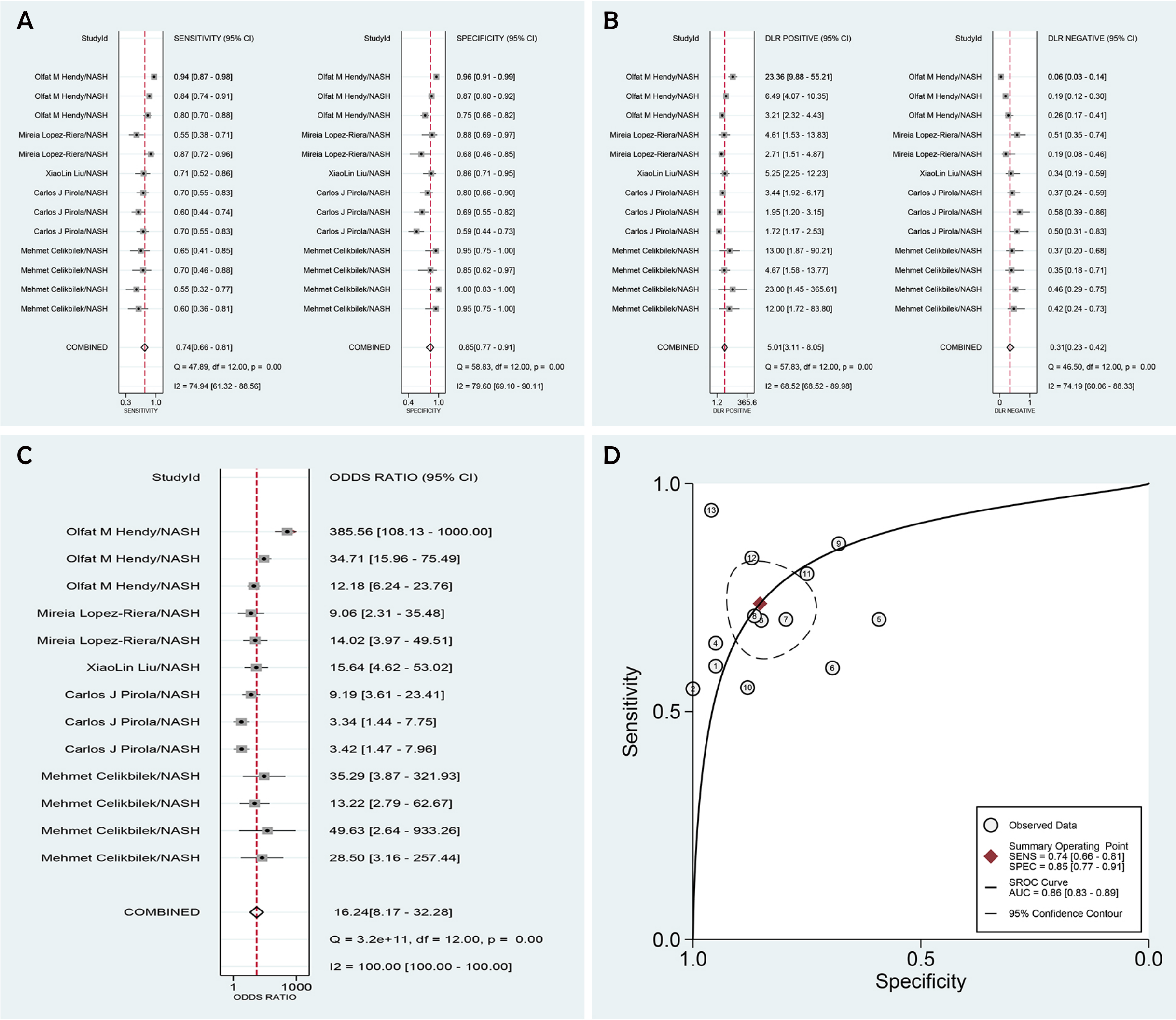
A
StudyId
SENSITIVITY (95% CI)
Olfat M Hendy/NASH 0.94 [0.87 - 0.98]
Olfat M Hendy/NASH 0.84 [0.74 - 0.91]
Olfat M Hendy/NASH 0.80 [0.70 - 0.88]
Mireia Lopez-Riera/NASH 0.55 [0.38 - 0.71]
Mireia Lopez-Riera/NASH 0.87 [0.72 - 0.96]
XiaoLin Liu/NASH 0.71 [0.52 - 0.86]
Carlos J Pirola/NASH 0.70 [0.55 - 0.83]
Carlos J Pirola/NASH 0.60 [0.44 - 0.74]
Carlos J Pirola/NASH 0.70 [0.55 - 0.83]
Mehmet Celikbilek/NASH 0.65 [0.41 - 0.85]
Mehmet Celikbilek/NASH 0.70 [0.46 - 0.88]
Mehmet Celikbilek/NASH 0.55 [0.32 - 0.77]
Mehmet Celikbilek/NASH 0.60 [0.36 - 0.81]
COMBINED 0.74[0.66 - 0.81]
Q = 47.89, df = 12.00, p = 0.00
I2 = 74.94 [61.32 - 88.56]
0.3 1.0
SENSITIVITY
StudyId
SPECIFICITY (95% CI)
Olfat M Hendy/NASH 0.96 [0.91 - 0.99]
Olfat M Hendy/NASH 0.87 [0.80 - 0.92]
Olfat M Hendy/NASH 0.75 [0.66 - 0.82]
Mireia Lopez-Riera/NASH 0.88 [0.69 - 0.97]
Mireia Lopez-Riera/NASH 0.68 [0.46 - 0.85]
XiaoLin Liu/NASH 0.86 [0.71 - 0.95]
Carlos J Pirola/NASH 0.80 [0.66 - 0.90]
Carlos J Pirola/NASH 0.69 [0.55 - 0.82]
Carlos J Pirola/NASH 0.59 [0.44 - 0.73]
Mehmet Celikbilek/NASH 0.95 [0.75 - 1.00]
Mehmet Celikbilek/NASH 0.85 [0.62 - 0.97]
Mehmet Celikbilek/NASH 1.00 [0.83 - 1.00]
Mehmet Celikbilek/NASH 0.95 [0.75 - 1.00]
COMBINED 0.85[0.77 - 0.91]
Q = 58.83, df = 12.00, p = 0.00
I2 = 79.60 [69.10 - 90.11]
0.4 1.0
SPECIFICITY
B
StudyId
DLR POSITIVE (95% CI)
Olfat M Hendy/NASH 23.36 [9.88 - 55.21]
Olfat M Hendy/NASH 6.49 [4.07 - 10.35]
Olfat M Hendy/NASH 3.21 [2.32 - 4.43]
Mireia Lopez-Riera/NASH 4.61 [1.53 - 13.83]
Mireia Lopez-Riera/NASH 2.71 [1.51 - 4.87]
XiaoLin Liu/NASH 5.25 [2.25 - 12.23]
Carlos J Pirola/NASH 3.44 [1.92 - 6.17]
Carlos J Pirola/NASH 1.95 [1.20 - 3.15]
Carlos J Pirola/NASH 1.72 [1.17 - 2.53]
Mehmet Celikbilek/NASH 13.00 [1.87 - 90.21]
Mehmet Celikbilek/NASH 4.67 [1.58 - 13.77]
Mehmet Celikbilek/NASH 23.00 [1.45 - 365.61]
Mehmet Celikbilek/NASH 12.00 [1.72 - 83.80]
COMBINED 5.01[3.11 - 8.05]
Q = 57.83, df = 12.00, p = 0.00
I2 = 68.52 [68.52 - 89.98]
1.2 365.6
DLR POSITIVE
StudyId
DLR NEGATIVE (95% CI)
Olfat M Hendy/NASH 0.06 [0.03 - 0.14]
Olfat M Hendy/NASH 0.19 [0.12 - 0.30]
Olfat M Hendy/NASH 0.26 [0.17 - 0.41]
Mireia Lopez-Riera/NASH 0.51 [0.35 - 0.74]
Mireia Lopez-Riera/NASH 0.19 [0.08 - 0.46]
XiaoLin Liu/NASH 0.34 [0.19 - 0.59]
Carlos J Pirola/NASH 0.37 [0.24 - 0.59]
Carlos J Pirola/NASH 0.58 [0.39 - 0.86]
Carlos J Pirola/NASH 0.50 [0.31 - 0.83]
Mehmet Celikbilek/NASH 0.37 [0.20 - 0.68]
Mehmet Celikbilek/NASH 0.35 [0.18 - 0.71]
Mehmet Celikbilek/NASH 0.46 [0.29 - 0.75]
Mehmet Celikbilek/NASH 0.42 [0.24 - 0.73]
COMBINED 0.31[0.23 - 0.42]
Q = 46.50, df = 12.00, p = 0.00
I2 = 74.19 [60.06 - 88.33]
0 1
DLR NEGATIVE
C
StudyId
ODDS RATIO (95% CI)
Olfat M Hendy/NASH 385.56 [108.13 - 1000.00]
Olfat M Hendy/NASH 34.71 [15.96 - 75.49]
Olfat M Hendy/NASH 12.18 [6.24 - 23.76]
Mireia Lopez-Riera/NASH 9.06 [2.31 - 35.48]
Mireia Lopez-Riera/NASH 14.02 [3.97 - 49.51]
XiaoLin Liu/NASH 15.64 [4.62 - 53.02]
Carlos J Pirola/NASH 9.19 [3.61 - 23.41]
Carlos J Pirola/NASH 3.34 [1.44 - 7.75]
Carlos J Pirola/NASH 3.42 [1.47 - 7.96]
Mehmet Celikbilek/NASH 35.29 [3.87 - 321.93]
Mehmet Celikbilek/NASH 13.22 [2.79 - 62.67]
Mehmet Celikbilek/NASH 49.63 [2.64 - 933.26]
Mehmet Celikbilek/NASH 28.50 [3.16 - 257.44]
COMBINED 16.24[8.17 - 32.28]
Q = 3.2e+11, df = 12.00, p = 0.00
I2 = 100.00 [100.00 - 100.00]
1 1000
ODDS RATIO
D
1.0
0.5
0.0
Sensitivity
1.0
0.5
0.0
Specificity
Observed Data
Summary Operating Point
SENS = 0.74 [0.66 - 0.81]
SPEC = 0.85 [0.77 - 0.91]
SROC Curve
AUC = 0.86 [0.83 - 0.89]
95% Confidence Contour

Figure S6

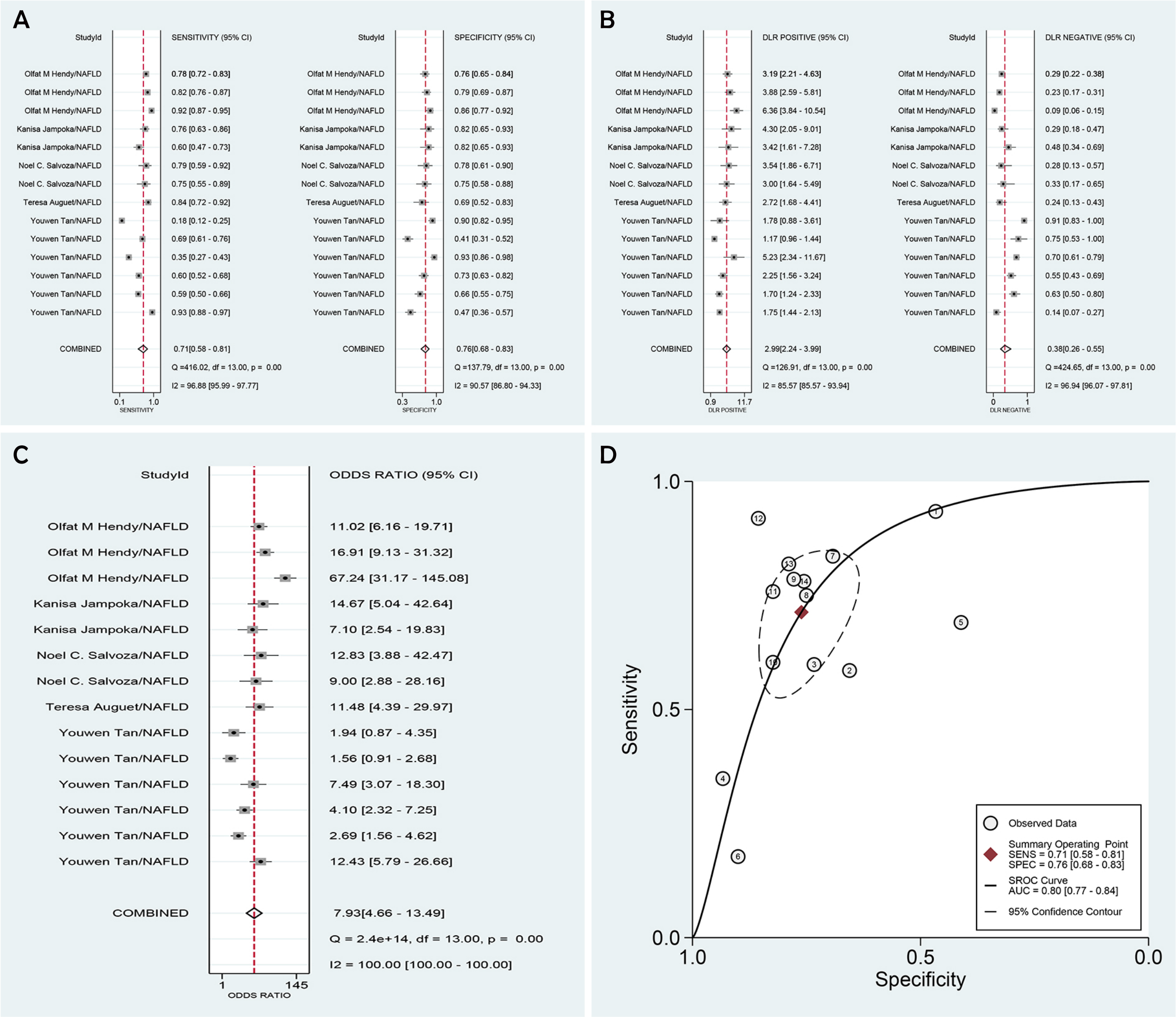
A
B
C
D
SENSITIVITY (95% CI)
SPECIFICITY (95% CI)
DLR POSITIVE (95% CI)
DLR NEGATIVE (95% CI)
ODDS RATIO (95% CI)
COMBINED
0.71[0.58 - 0.81]
0.76[0.68 - 0.83]
2.99[2.24 - 3.99]
0.38[0.26 - 0.55]
7.93[4.66 - 13.49]
Q = 2.4e+14, df = 13.00, p = 0.00
I2 = 100.00 [100.00 - 100.00]
Sensitivity
Specificity
Observed Data
Summary Operating Point
SENS = 0.71 [0.58 - 0.81]
SPEC = 0.76 [0.68 - 0.83]
SROC Curve
AUC = 0.80 [0.77 - 0.84]
95% Confidence Contour

Figure S7

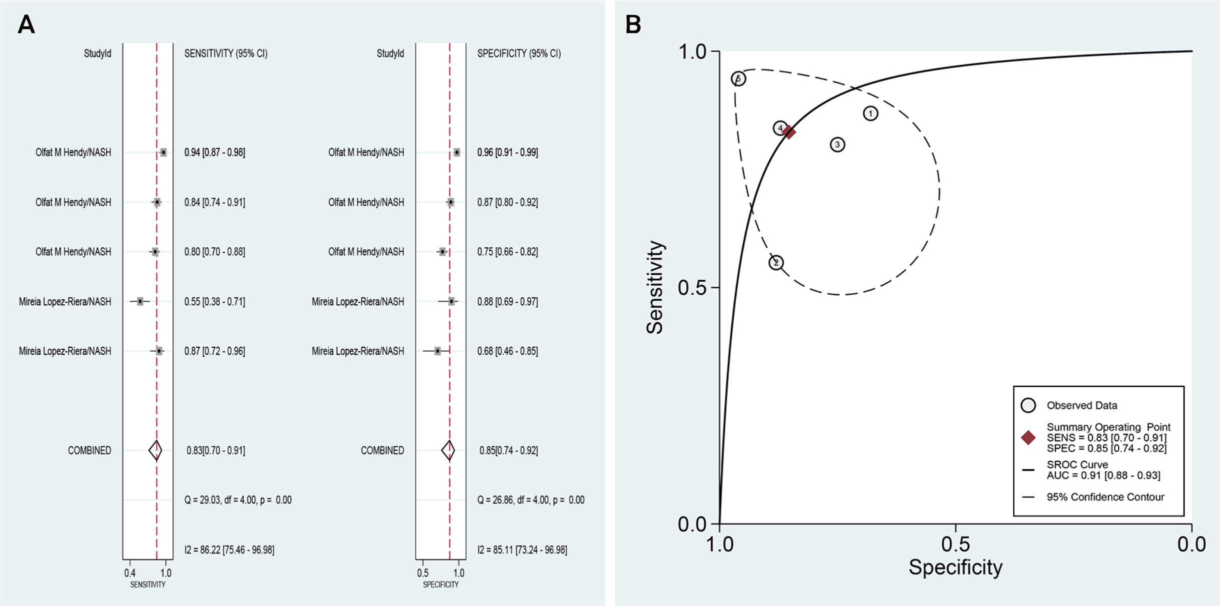
A
StudyId
SENSITIVITY (95% CI)
Olfat M Hendy/NASH 0.94 [0.87 - 0.98]
Olfat M Hendy/NASH 0.84 [0.74 - 0.91]
Olfat M Hendy/NASH 0.80 [0.70 - 0.88]
Mireia Lopez-Riera/NASH 0.55 [0.38 - 0.71]
Mireia Lopez-Riera/NASH 0.87 [0.72 - 0.96]
COMBINED 0.83[0.70 - 0.91]
Q = 29.03, df = 4.00, p = 0.00
I2 = 86.22 [75.46 - 96.98]
0.4
1.0
SENSITIVITY
StudyId
SPECIFICITY (95% CI)
Olfat M Hendy/NASH 0.96 [0.91 - 0.99]
Olfat M Hendy/NASH 0.87 [0.80 - 0.92]
Olfat M Hendy/NASH 0.75 [0.66 - 0.82]
Mireia Lopez-Riera/NASH 0.88 [0.69 - 0.97]
Mireia Lopez-Riera/NASH 0.68 [0.46 - 0.85]
COMBINED 0.85[0.74 - 0.92]
Q = 26.86, df = 4.00, p = 0.00
I2 = 85.11 [73.24 - 96.98]
0.5
1.0
SPECIFICITY
B
1.0
0.5
0.0
Sensitivity
1.0
0.5
0.0
Specificity
Observed Data
Summary Operating Point
SENS = 0.83 [0.70 - 0.91]
SPEC = 0.85 [0.74 - 0.92]
SROC Curve
AUC = 0.91 [0.88 - 0.93]
95% Confidence Contour

Table S1. The characteristic of the included trials

Trial number	First author	Year	Geographic region	Target microRNAs	Regulation mode	Disease	Sample size (*n*)		BMI (kg/m^2)	Proportion of males (%)	Diagnostic power	
							Control (healthy/NAFL)	Case			Sensitivity (%)	Specificity (%)
							Control (NAS < 5)	NASH (NAS ≥ 5)				
1	Mehmet Celikbilek	2014	Turkey	miR-197	Downregulation	NASH	20/0	20	31.86	18 (45%)	60	95
2	Mehmet Celikbilek	2014	Turkey	miR-146b	Downregulation	NASH	20/0	20	31.86	18 (45%)	55	100
3	Mehmet Celikbilek	2014	Turkey	miR-181d	Downregulation	NASH	20/0	20	31.86	18 (45%)	70	85
4	Mehmet Celikbilek	2014	Turkey	miR-99a	Downregulation	NASH	20/0	20	31.86	18 (45%)	65	96
5	Carlos J Pirola	2015	Argentina	miR-122	Upregulation	NASH	19/30	47	33.3	38 (57.6%)	70	60
6	Carlos J Pirola	2015	Argentina	miR-192	Upregulation	NASH	19/30	47	33.3	38 (57.6%)	60	70
7	Carlos J Pirola	2015	Argentina	miR-375	Upregulation	NASH	19/30	47	33.3	38 (57.6%)	70	80
8	XiaoLin Liu	2016	China	miR-34a	Upregulation	NASH	37/0	31	27.2	40 (58.8)	70.4	87.5
9	Mireia Lopez-Riera	2018	Spain	miR-192/miR-30C	Upregulation	NASH	0/25	38	32.4	—	87	69
10	Mireia Lopez-Riera	2018	Spain	miR-27b/miR-30C	Upregulation	NASH	0/25	38	32.4	—	55	89
11	Olfat M Hendy	2019	Egypt	miR-122	Upregulation	NASH	0/124	86	32.4	—	80	75
12	Olfat M Hendy	2019	Egypt	miR-34a	Upregulation	NASH	0/124	86	32.4	—	84	87
13	Olfat M Hendy	2019	Egypt	miR-99a	Downregulation	NASH	0/124	86	32.4	—	94	96
							Control (healthy)	NAFLD				
14	Youwen Tan	2014	China	miR-122-5p	Upregulation	NAFLD	90/0	152	24.74	195 (80.5%)	93.4	46.7
15	Youwen Tan	2014	China	miR-27b-3p	Upregulation	NAFLD	90/0	152	24.74	195 (80.5%)	58.6	65.6
16	Youwen Tan	2014	China	miR-192-5p	Upregulation	NAFLD	90/0	152	24.74	195 (80.5%)	59.9	72.7
17	Youwen Tan	2014	China	miR-148a-3p	Upregulation	NAFLD	90/0	152	24.74	195 (80.5%)	34.9	93.3
18	Youwen Tan	2014	China	miR-99a-5p	Upregulation	NAFLD	90/0	152	24.74	195 (80.5%)	69.1	41.1
19	Youwen Tan	2014	China	6 miRs panel	Upregulation	NAFLD	90/0	152	24.74	195 (80.5%)	25.7	90
20	Teresa Auguet	2016	Spain	miR-122	Upregulation	NAFLD	39/0	61	—	0 (0%)	83.1	69.8
21	Noel C. Salvoza	2016	Philippines	miR-34a	Upregulation	NAFLD	36/0	28	28.5	35 (54.7%)	75	75
22	Noel C. Salvoza	2016	Philippines	miR-122	Upregulation	NAFLD	36/0	28	28.5	35 (54.7%)	78.6	77.8
23	Kanisa Jampoka	2018	Thailand	miR-29a	Downregulation	NAFLD	34/0	58	—	—	60.37	82.35
24	Kanisa Jampoka	2018	Thailand	miR-122	Upregulation	NAFLD	34/0	58	—	—	75	82.35
25	Olfat M Hendy	2019	Egypt	miR-122	Upregulation	NAFLD	90/0	210	32.4	127（42.9%）	92	85
26	Olfat M Hendy	2019	Egypt	miR-34a	Upregulation	NAFLD	90/0	210	32.4	127（42.9%）	82	79
27	Olfat M Hendy	2019	Egypt	miR-99a	Downregulation	NAFLD	90/0	210	32.4	127（42.9%）	78	76

BMI: body mass index; NAFL: nonalcoholic fatty liver (simple steatosis); NAFLD: nonalcoholic fatty liver disease; NAS: NAFLD activity score; NASH: nonalcoholic steatohepatitis

Table S2. Heterogeneity of serum miRNA for diagnosing total NAFLD after omitting the trials with study factors

Study factors for omitting	Pooled values after factors omitted			
	Sensitivity (95% CI)	I^2 (%)	Specificity (95% CI)	I^2 (%)
—	0.72 (0.64, 0.79)	94.82	0.81 (0.75, 0.86)	88.37
BMI < 30 kg/m^2	0.77(0.70, 0.82)	81.28	0.83 (0.77, 0.87)	72.92
BMI < 30 kg/m^2+ proportion of males (%) ⩾ 50	0.76 (0.65, 0.84)	80.39	0.90 (0.76, 0.93)	76.92
BMI < 30 kg/m^2 + proportion of males (%) ⩾ 50 + NAFLD trials +upregulation mode	0.63 (0.51, 0.73)	0	0.94 (0.84, 0.98)	25.99

BMI: body mass index; CI: confidence interval; NAFLD: nonalcoholic fatty liver disease

PRISMA 2009 Checklist

Section/topic	#	Checklist item	Reported on page #
**TITLE**			
Title	1	Identify the report as a systematic review, meta-analysis, or both.	1
**ABSTRACT**			
Structured summary	2	Provide a structured summary including, as applicable: background; objectives; data sources; study eligibility criteria, participants, and interventions; study appraisal and synthesis methods; results; limitations; conclusions and implications of key findings; systematic review registration number.	2
**INTRODUCTION**			
Rationale	3	Describe the rationale for the review in the context of what is already known.	3
Objectives	4	Provide an explicit statement of questions being addressed with reference to participants, interventions, comparisons, outcomes, and study design (PICOS).	4
**METHODS**			
Protocol and registration	5	Indicate if a review protocol exists, if and where it can be accessed (e.g., Web address), and, if available, provide registration information including registration number.	5
Eligibility criteria	6	Specify study characteristics (e.g., PICOS, length of follow-up) and report characteristics (e.g., years considered, language, publication status) used as criteria for eligibility, giving rationale.	5
Information sources	7	Describe all information sources (e.g., databases with dates of coverage, contact with study authors to identify additional studies) in the search and date last searched.	5
Search	8	Present full electronic search strategy for at least one database, including any limits used, such that it could be repeated.	5
Study selection	9	State the process for selecting studies (i.e., screening, eligibility, included in systematic review, and, if applicable, included in the meta-analysis).	5
Data collection process	10	Describe method of data extraction from reports (e.g., piloted forms, independently, in duplicate) and any processes for obtaining and confirming data from investigators.	5-6
Data items	11	List and define all variables for which data were sought (e.g., PICOS, funding sources) and any assumptions and simplifications made.	6
Risk of bias in individual studies	12	Describe methods used for assessing risk of bias of individual studies (including specification of whether this was done at the study or outcome level), and how this information is to be used in any data synthesis.	NA
Summary measures	13	State the principal summary measures (e.g., risk ratio, difference in means).	6
Synthesis of results	14	Describe the methods of handling data and combining results of studies, if done, including measures of consistency (e.g., I^2) for each meta-analysis.	6-7

PRISMA 2009 Checklist

Section/topic	#	Checklist item	Reported on page #
Risk of bias across studies	15	Specify any assessment of risk of bias that may affect the cumulative evidence (e.g., publication bias, selective reporting within studies).	6-7
Additional analyses	16	Describe methods of additional analyses (e.g., sensitivity or subgroup analyses, meta-regression), if done, indicating which were pre-specified.	6-7
**RESULTS**			
Study selection	17	Give numbers of studies screened, assessed for eligibility, and included in the review, with reasons for exclusions at each stage, ideally with a flow diagram.	7
Study characteristics	18	For each study, present characteristics for which data were extracted (e.g., study size, PICOS, follow-up period) and provide the citations.	7-8
Risk of bias within studies	19	Present data on risk of bias of each study and, if available, any outcome level assessment (see item 12).	NA
Results of individual studies	20	For all outcomes considered (benefits or harms), present, for each study: (a) simple summary data for each intervention group (b) effect estimates and confidence intervals, ideally with a forest plot.	8-9
Synthesis of results	21	Present results of each meta-analysis done, including confidence intervals and measures of consistency.	8-11
Risk of bias across studies	22	Present results of any assessment of risk of bias across studies (see Item 15).	12-13
Additional analysis	23	Give results of additional analyses, if done (e.g., sensitivity or subgroup analyses, meta-regression [see Item 16]).	10-12
**DISCUSSION**			
Summary of evidence	24	Summarize the main findings including the strength of evidence for each main outcome; consider their relevance to key groups (e.g., healthcare providers, users, and policy makers).	13
Limitations	25	Discuss limitations at study and outcome level (e.g., risk of bias), and at review-level (e.g., incomplete retrieval of identified research, reporting bias).	18-19
Conclusions	26	Provide a general interpretation of the results in the context of other evidence, and implications for future research.	19
**FUNDING**			
Funding	27	Describe sources of funding for the systematic review and other support (e.g., supply of data); role of funders for the systematic review.	22

From: Moher D, Liberati A, Tetzlaff J, Altman DG, The PRISMA Group (2009). Preferred Reporting Items for Systematic Reviews and Meta-Analyses: The PRISMA Statement. PLoS Med 6(6): e1000097. doi:10.1371/journal.pmed1000097

For more information, visit: www.prisma-statement.org.
